# Death in hospital following ICU discharge: insights from the LUNG SAFE study

**DOI:** 10.1186/s13054-021-03465-0

**Published:** 2021-04-13

**Authors:** Fabiana Madotto, Bairbre McNicholas, Emanuele Rezoagli, Tài Pham, John G. Laffey, Giacomo Bellani, the LUNG SAFE Investigators, Antonio Pesenti, John G. Laffey, Laurent Brochard, Andres Esteban, Luciano Gattinoni, Frank van Haren, Marco Ranieri, Gordon Rubenfeld, Gordon Rubenfeld, B. Taylor Thompson, Arthur S. Slutsky, Fernando Rios, Frank van Haren, Mohammad Omar Faruq, T. Sottiaux, P. Depuydt, Fredy S. Lora, Cesar Cesar Azevedo, Eddy Fan, Guillermo Bugedo, Haibo Qiu, Marcos Gonzalez, Juan Silesky, Vladimir Cerny, Jonas Nielsen, Manuel Jibaja, Tài Pham, Hermann Wrigge, Dimitrios Matamis, Jorge Luis Ranero, Charles Gomersall, Pravin Amin, S. M. Hashemian, Kevin Clarkson, Giacomo Bellani, Kiyoyasu Kurahashi, Younsuck Koh, Asisclo Villagomez, Amine Ali Zeggwagh, Leo M. Heunks, Jon Henrik Laake, Waqar Kashif, Jorge Synclair, Jose Emmanuel Palo, Antero do Vale Fernandes, Dorel Sandesc, Yaasen Arabi, Vesna Bumbasierevic, Nicolas Nin, Jose A. Lorente, Anders Larsson, Lise Piquilloud, Boonsong Patjanasoontorn, Fekri Abroug, Daniel F. McAuley, Lia McNamee, Javier Hurtado, Ed Bajwa, Gabriel Démpaire, Guy M. Francois, Francesca Rabboni, Fabiana Madotto, Sara Conti, Hektor Sula, Lordian Nunci, Alma Cani, Alan Zazu, Christian Dellera, Carolina S. Insaurralde, Risso V. Alejandro, Julio Daldin, Mauricio Vinzio, Ruben O. Fernandez, Luis P. Cardonnet, Lisandro R. Bettini, Mariano Carboni Bisso, Emilio M. Osman, Mariano G. Setten, Pablo Lovazzano, Javier Alvarez, Veronica Villar, Norberto C. Pozo, Nicolas Grubissich, Gustavo A. Plotnikow, Daniela N. Vasquez, Santiago Ilutovich, Norberto Tiribelli, Ariel Chena, Carlos A. Pellegrini, María G. Saenz, Elisa Estenssoro, Matias Brizuela, Hernan Gianinetto, Pablo E. Gomez, Valeria I. Cerrato, Marco G. Bezzi, Silvina A. Borello, Flavia A. Loiacono, Adriana M. Fernandez, Serena Knowles, Claire Reynolds, Deborah M. Inskip, Jennene J. Miller, Jing Kong, Christina Whitehead, Shailesh Bihari, Aylin Seven, Amanda Krstevski, Helen J. Rodgers, Rebecca T. Millar, Toni E. Mckenna, Irene M. Bailey, Gabrielle C. Hanlon, Anders Aneman, Joan M. Lynch, Raman Azad, John Neal, Paul W. Woods, Brigit L. Roberts, Mark R. Kol, Helen S. Wong, Katharina C. Riss, Thomas Staudinger, Xavier Wittebole, Caroline Berghe, Pierre A. Bulpa, Alain M. Dive, Rik Verstraete, Herve Lebbinck, Pieter Depuydt, Joris Vermassen, Philippe Meersseman, Helga Ceunen, Jonas I. Rosa, Daniel O. Beraldo, Claudio Piras, Adenilton M. Rampinelli, ñ P. Nassar, Sergio Mataloun, Marcelo Moock, Marlus M. Thompson, Claudio H. Gonçalves, Ana Carolina P. Antônio, Aline Ascoli, Rodrigo S. Biondi, Danielle C. Fontenele, Danielle Nobrega, Vanessa M. Sales, Suresh Shindhe, Dk Maizatul Aiman, BPg Hj Ismail, John Laffey, Francois Beloncle, Kyle G. Davies, Rob Cirone, Venika Manoharan, Mehvish Ismail, Ewan C. Goligher, Mandeep Jassal, Niall D. Ferguson, Erin Nishikawa, Areej Javeed, Gerard Curley, Nuttapol Rittayamai, Matteo Parotto, Sangeeta Mehta, Jenny Knoll, Antoine Pronovost, Sergio Canestrini, Alejandro R. Bruhn, Patricio H. Garcia, Felipe A. Aliaga, Pamela A. Farías, Jacob S. Yumha, Claudia A. Ortiz, Javier E. Salas, Alejandro A. Saez, Luis D. Vega, Eduardo F. Labarca, Felipe T. Martinez, Nicolás G. Carreño, Pilar Lora, Haitao Liu, Haibo Qiu, Ling Liu, Rui Tang, Xiaoming Luo, Youzhong An, Huiying Zhao, Yan Gao, Zhe Zhai, Zheng L. Ye, Wei Wang, Wenwen Li, Qingdong Li, Ruiqiang Zheng, Wenkui Yu, Juanhong Shen, Xinyu Li, Tao Yu, Weihua Lu, Ya Q. Wu, Xiao B. Huang, Zhenyang He, Yuanhua Lu, Hui Han, Fan Zhang, Renhua Sun, Hua X. Wang, Shu H. Qin, Bao H. Zhu, Jun Zhao, Jian Liu, Bin Li, Jing L. Liu, Fa C. Zhou, Qiong J. Li, Xing Y. Zhang, Zhou Li-Xin, Qiang Xin-Hua, Liangyan Jiang, Yuan N. Gao, Xian Y. Zhao, Yuan Y. Li, Xiao L. Li, Chunting Wang, Qingchun Yao, Rongguo Yu, Kai Chen, Huanzhang Shao, Bingyu Qin, Qing Q. Huang, Wei H. Zhu, Ai Y. Hang, Ma X. Hua, Yimin Li, Yonghao Xu, Yu D. Di, Long L. Ling, Tie H. Qin, Shou H. Wang, Junping Qin, Yi Han, Suming Zhou, Monica P. Vargas, Juan I. Silesky Jimenez, Manuel A. González Rojas, Jaime E. Solis-Quesada, Christian M. Ramirez-Alfaro, Jan Máca, Peter Sklienka, Jakob Gjedsted, Aage Christiansen, Jonas Nielsen, Boris G. Villamagua, Miguel Llano, Philippe Burtin, Gautier Buzancais, Pascal Beuret, Nicolas Pelletier, Satar Mortaza, Alain Mercat, Jonathan Chelly, Sébastien Jochmans, Nicolas Terzi, Cédric Daubin, Guillaume Carteaux, Nicolas de Prost, Jean-Daniel Chiche, Fabrice Daviaud, Tài Pham, Muriel Fartoukh, Guillaume Barberet, Jerome Biehler, Jean Dellamonica, Denis Doyen, Jean-Michel Arnal, Anais Briquet, Fanny Klasen, Laurent Papazian, Arnaud Follin, Damien Roux, Jonathan Messika, Evangelos Kalaitzis, Laurence Dangers, Alain Combes, Siu-Ming Au, Gaetan Béduneau, Dorothée Carpentier, Elie H. Zogheib, Herve Dupont, Sylvie Ricome, Francesco L. Santoli, Sebastien L. Besset, Philippe Michel, Bruno Gelée, Pierre-Eric Danin, Bernard Goubaux, Philippe J. Crova, Nga T. Phan, Frantz Berkelmans, Julio C. Badie, Romain Tapponnier, Josette Gally, Samy Khebbeb, Jean-Etienne Herbrecht, Francis Schneider, Pierre-Louis M. Declercq, Jean-Philippe Rigaud, Jacques Duranteau, Anatole Harrois, Russell Chabanne, Julien Marin, Jean-Michel Constantin, Sandrine Thibault, Mohammed Ghazi, Messabi Boukhazna, Salem Ould Zein, Jack R. Richecoeur, Daniele M. Combaux, Fabien Grelon, Charlene Le Moal, Elise P. Sauvadet, Adrien Robine, Virginie Lemiale, Danielle Reuter, Martin Dres, Alexandre Demoule, Dany Goldgran-Toledano, Loredana Baboi, Claude Guérin, Ralph Lohner, Jens Kraßler, Susanne Schäfer, Kai D. Zacharowski, Patrick Meybohm, Andreas W. Reske, Philipp Simon, Hans-Bernd F. Hopf, Michael Schuetz, Thomas Baltus, Metaxia N. Papanikolaou, Theonymfi G. Papavasilopoulou, Giannis A. Zacharas, Vasilis Ourailogloy, Eleni K. Mouloudi, Eleni V. Massa, Eva O. Nagy, Electra E. Stamou, Ellada V. Kiourtzieva, Marina A. Oikonomou, Luis E. Avila, Cesar A. Cortez, Johanna E. Citalán, Sameer A. Jog, Safal D. Sable, Bhagyesh Shah, Mohan Gurjar, Arvind K. Baronia, Mohammedfaruk Memon, Radhakrishnan Muthuchellappan, Venkatapura J. Ramesh, Anitha Shenoy, Ramesh Unnikrishnan, Subhal B. Dixit, Rachana V. Rhayakar, Nagarajan Ramakrishnan, Vallish K. Bhardwaj, Heera L. Mahto, Sudha V. Sagar, Vijayanand Palaniswamy, Deeban Ganesan, Seyed Mohammadreza Hashemian, Hamidreza Jamaati, Farshad Heidari, Edel A. Meaney, Alistair Nichol, Karl M. Knapman, Donall O’Croinin, Eimhin S. Dunne, Dorothy M. Breen, Kevin P. Clarkson, Rola F. Jaafar, Rory Dwyer, Fahd Amir, Olaitan O. Ajetunmobi, Aogan C. O’Muircheartaigh, Colin S. Black, Nuala Treanor, Daniel V. Collins, Wahid Altaf, Gianluca Zani, Maurizio Fusari, Savino Spadaro, Carlo A. Volta, Romano Graziani, Barbara Brunettini, Salvatore Palmese, Paolo Formenti, Michele Umbrello, Andrea Lombardo, Elisabetta Pecci, Marco Botteri, Monica Savioli, Alessandro Protti, Alessia Mattei, Lorenzo Schiavoni, Andrea Tinnirello, Manuel Todeschini, Policlinico P. Giaccone, Antonino Giarratano, Andrea Cortegiani, Sara Sher, Anna Rossi, Massimo M. Antonelli, Luca M. Montini, Paolo Casalena, Sergio Scafetti, Giovanna Panarello, Giovanna Occhipinti, Nicolò Patroniti, Matteo Pozzi, Roberto R. Biscione, Michela M. Poli, Ferdinando Raimondi, Daniela Albiero, Giulia Crapelli, Eduardo Beck, Vincenzo Pota, Vincenzo Schiavone, Alexandre Molin, Fabio Tarantino, Giacomo Monti, Elena Frati, Lucia Mirabella, Gilda Cinnella, Tommaso Fossali, Riccardo Colombo, Pierpaolo Terragni Ilaria Pattarino, Francesco Mojoli, Antonio Braschi, Erika E. Borotto, Andrea N. Cracchiolo, Daniela M. Palma, Francesco Raponi, Giuseppe Foti, Ettore R. Vascotto, Andrea Coppadoro, Luca Brazzi, Leda Floris, Giorgio A. Iotti, Aaron Venti, Osamu Yamaguchi, Shunsuke Takagi, Hiroki N. Maeyama, Eizo Watanabe, Yoshihiro Yamaji, Kazuyoshi Shimizu, Kyoko Shiozaki, Satoru Futami, Sekine Ryosuke, Koji Saito, Yoshinobu Kameyama, Keiko Ueno, Masayo Izawa, Nao Okuda, Hiroyuki Suzuki, Tomofumi Harasawa, Michitaka Nasu, Tadaaki Takada, Fumihito Ito, Shin Nunomiya, Kansuke Koyama, Toshikazu Abe, Kohkichi Andoh, Kohei Kusumoto, Akira Hirata, Akihiro Takaba, Hiroyasu Kimura, Shuhei Matsumoto, Ushio Higashijima, Hiroyuki Honda, Nobumasa Aoki, Hiroshi Imai, Yasuaki Ogino, Ichiko Mizuguchi, Kazuya Ichikado, Kenichi Nitta, Katsunori Mochizuki, Tomoaki Hashida, Hiroyuki Tanaka, Tomoyuki Nakamura, Daisuke Niimi, Takeshi Ueda, Yozo Kashiwa, Akinori Uchiyama, Olegs Sabelnikovs, Peteris Oss, Youssef Haddad, Kong Y. Liew, Silvio A. Ñamendys-Silva, Yves D. Jarquin-Badiola, Luis A. Sanchez-Hurtado, Saira S. Gomez-Flores, Maria C. Marin, Asisclo J. Villagomez, Jordana S. Lemus, Jonathan M. Fierro, Mavy Ramirez Cervantes, Francisco Javier Flores Mejia, Dulce Dector, Dulce M. Dector, Daniel R. Gonzalez, Claudia R. Estrella, Jorge R. Sanchez-Medina, Alvaro Ramirez-Gutierrez, Fernando G. George, Janet S. Aguirre, Juan A. Buensuseso, Manuel Poblano, Mohammed V. University, Tarek Dendane, Amine Ali Zeggwagh, Hicham Balkhi, Mina Elkhayari, Nacer Samkaoui, Hanane Ezzouine, Abdellatif Benslama, Mourad Amor, Wajdi Maazouzi, Nedim Cimic, Oliver Beck, Monique M. Bruns, Jeroen A. Schouten, Myra Rinia, Monique Raaijmakers, Leo M. Heunks, Hellen M. Van Wezel, Serge J. Heines, Ulrich Strauch, Marc P. Buise, Fabienne D. Simonis, Marcus J. Schultz, Jennifer C. Goodson, Troy S. Browne, Leanlove Navarra, Anna Hunt, Robyn A. Hutchison, Mathew B. Bailey, Lynette Newby, Colin Mcarthur, Michael Kalkoff, Alex Mcleod, Jonathan Casement, Danielle J. Hacking, Finn H. Andersen, Merete S. Dolva, Jon H. Laake, Andreas Barratt-Due, Kim Andre L. Noremark, Eldar Søreide, Brit Å. Sjøbø, Anne B. Guttormsen, Hector H. Leon Yoshido, Ronald Zumaran Aguilar, Fredy A. Montes Oscanoa, Alain U. Alisasis, Joanne B. Robles, Rossini Abbie B. Pasanting-Lim, Beatriz C. Tan, Pawel Andruszkiewicz, Karina Jakubowska, Cristina M. Coxo, António M. Alvarez, Bruno S. Oliveira, Gustavo M. Montanha, Nelson C. Barros, Carlos S. Pereira, António M. Messias, Jorge M. Monteiro, Ana M. Araujo, Nuno T. Catorze, Susan M. Marum, Maria J. Bouw, Rui M. Gomes, Vania A. Brito, Silvia Castro, Joana M. Estilita, Filipa M. Barros, Isabel M. Serra, Aurelia M. Martinho, Dana R. Tomescu, Alexandra Marcu, Ovidiu H. Bedreag, Marius Papurica, Dan E. Corneci, Silvius Ioan Negoita, Evgeny Grigoriev, Alexey I. Gritsan, Andrey A. Gazenkampf, Ghaleb Almekhlafi, Mohamad M. Albarrak, Ghanem M. Mustafa, Khalid A. Maghrabi, Nawal Salahuddin, Tharwat M. Aisa, Ahmed S. Al Jabbary, Edgardo Tabhan, Yaseen M. Arabi, Olivia A. Trinidad, Hasan M. Al Dorzi, Edgardo E. Tabhan, Stefan Bolon, Oliver Smith, Jordi Mancebo, Hernan Aguirre-Bermeo, Juan C. Lopez-Delgado, Francisco Esteve, Gemma Rialp, Catalina Forteza, Candelaria De Haro, Antonio Artigas, Guillermo M. Albaiceta, Sara De Cima-Iglesias, Leticia Seoane-Quiroga, Alexandra Ceniceros-Barros, Antonio L. Ruiz-Aguilar, Luis M. Claraco-Vega, Juan Alfonso Soler, Maria del Carmen Lorente, Cecilia Hermosa, Federico Gordo, Miryam Prieto-González, Juan B. López-Messa, Manuel P. Perez, Cesar P. Perez, Raquel Montoiro Allue, Ferran Roche-Campo, Marcos Ibañez-Santacruz, Susana Temprano, Maria C. Pintado, Raul De Pablo, Pilar Ricart Aroa Gómez, Silvia Rodriguez Ruiz, Silvia Iglesias Moles, Mª Teresa Jurado, Alfons Arizmendi, Enrique A. Piacentini, Nieves Franco, Teresa Honrubia, Meisy Perez Cheng, Elena Perez Losada, Javier Blanco, Luis J. Yuste, Cecilia Carbayo-Gorriz, Francisca G. Cazorla-Barranquero, Javier G. Alonso, Rosa S. Alda, Ángela Algaba, Gonzalo Navarro, Enrique Cereijo, Esther Diaz-Rodriguez, Diego Pastor Marcos, Laura Alvarez Montero, Luis Herrera Para, Roberto Jimenez Sanchez, Miguel Angel Blasco Navalpotro, Ricardo Diaz Abad, Raquel Montiel Gonz á lez, Dácil Parrilla Toribio, Alejandro G. Castro, Maria Jose D. Artiga, Oscar Penuelas, Tomas P. Roser, Moreno F. Olga, Elena Gallego Curto, Rocío Manzano Sánchez, Vallverdu P. Imma, Garcia M. Elisabet, Laura Claverias, Monica Magret, Ana M. Pellicer, Lucia L. Rodriguez, Jesús Sánchez-Ballesteros, Ángela González-Salamanca, Antonio G. Jimenez, Francisco P. Huerta, Juan Carlos J. Sotillo Diaz, Esther Bermejo Lopez, David D. Llinares Moya, Alec A. Tallet Alfonso, Palazon Sanchez Eugenio Luis, Palazon Sanchez Cesar, Sánchez I. Rafael, Corcoles G. Virgilio, Noelia N. Recio, Richard O. Adamsson, Christian C. Rylander, Bernhard Holzgraefe, Lars M. Broman, Joanna Wessbergh, Linnea Persson, Fredrik Schiöler, Hans Kedelv, Anna Oscarsson Tibblin, Henrik Appelberg, Lars Hedlund, Johan Helleberg, Karin E. Eriksson, Rita Glietsch, Niklas Larsson, Ingela Nygren, Silvia L. Nunes, Anna-Karin Morin, Thomas Kander, Anne Adolfsson, Lise Piquilloud, Hervé O. Zender, Corinne Leemann-Refondini, Souheil Elatrous, Slaheddine Bouchoucha, Imed Chouchene, Islem Ouanes, La Marsa, Asma Ben Souissi, Salma Kamoun, Oktay Demirkiran, Mustafa Aker, Emre Erbabacan, Ilkay Ceylan, Nermin Kelebek Girgin, Menekse Ozcelik, Necmettin Ünal, Basak Ceyda Meco, Onat O. Akyol, Suleyman S. Derman, Barry Kennedy, Ken Parhar, Latha Srinivasa, Lia McNamee, Danny McAuley, Phil Hopkins, Clare Mellis, Vivek Kakar, Dan Hadfield, Andre Vercueil, Kaushik Bhowmick, Sally K. Humphreys, Andrew Ferguson, Raymond Mckee, Ashok S. Raj, Danielle A. Fawkes, Philip Watt, Linda Twohey, Rajeev R. JhaMatthew Thomas, Alex Morton, Varsha Kadaba, Mark J. Smith, Anil P. Hormis, Santhana G. Kannan, Miriam Namih, Henrik Reschreiter, Julie Camsooksai, Alek Kumar, Szabolcs Rugonfalvi, Christopher Nutt, Orla Oneill, Colette Seasman, Ged Dempsey, Christopher J. Scott, Helen E. Ellis, Stuart Mckechnie, Paula J. Hutton, Nora N. Di Tomasso, Michela N. Vitale, Ruth O. Griffin, Michael N. Dean, Julius H. Cranshaw, Emma L. Willett, Nicholas Ioannou, Sarah Gillis, Peter Csabi, Rosaleen Macfadyen, Heidi Dawson, Pieter D. Preez, Alexandra J. Williams, Owen Boyd, Laura Ortiz-Ruiz De Gordoa, Jon Bramall, Sophie Symmonds, Simon K. Chau, Tim Wenham, Tamas Szakmany, Piroska Toth-Tarsoly, Katie H. Mccalman, Peter Alexander, Lorraine Stephenson, Thomas Collyer, Rhiannon Chapman, Raphael Cooper, Russell M. Allan, Malcolm Sim, David W. Wrathall, Donald A. Irvine, Kim S. Zantua, John C. Adams, Andrew J. Burtenshaw, Gareth P. Sellors, Ingeborg D. Welters, Karen E. Williams, Robert J. Hessell, Matthew G. Oldroyd, Ceri E. Battle, Suresh Pillai, Istvan Kajtor, Mageswaran Sivashanmugavel, Sinead C. Okane, Adrian Donnelly, Aniko D. Frigyik, Jon P. Careless, Martin M. May, Richard Stewart, T. John Trinder, Samantha J. Hagan, Matt P. Wise, Jade M. Cole, Caroline C. MacFie, Anna T. Dowling, Javier Hurtado, Nicolás Nin, Javier Hurtado, Edgardo Nuñez, Gustavo Pittini, Ruben Rodriguez, María C. Imperio, Cristina Santos, Alberto Deicas, Carolina Serra, Aditya Uppalapati, Ghassan Kamel, Valerie M. Banner-Goodspeed, Jeremy R. Beitler, Satyanarayana Reddy Mukkera, Shreedhar Kulkarni, Jarone Lee, Tomaz Mesar, John O. Shinn Iii, Dina Gomaa, Christopher Tainter, Jarone Lee, Tomaz Mesar, Jarone Lee, Dale J. Yeatts, Jessica Warren, Michael J. Lanspa, Russel R. Miller, Colin K. Grissom, Samuel M. Brown, Philippe R. Bauer, Ryan J. Gosselin, Barrett T. Kitch, Jason E. Cohen, Scott H. Beegle, Renaud M. Gueret, Aiman Tulaimat, Shazia Choudry, William Stigler, Hitesh Batra, Nidhi G. Huff, Keith D. Lamb, Trevor W. Oetting, Nicholas M. Mohr, Claine Judy, Shigeki Saito, Fayez M. Kheir, Fayez Kheir, Adam B. Schlichting, Angela Delsing, Daniel R. Crouch, Mary Elmasri, Daniel R. Crouch, Dina Ismail, Kyle R. Dreyer, Thomas C. Blakeman, Kyle R. Dreyer, Dina Gomaa, Rebecca M. Baron, Carolina Quintana Grijalba, Peter C. Hou, Raghu Seethala, Imo Aisiku, Galen Henderson, Gyorgy Frendl, Sen-Kuang Hou, Robert L. Owens, Ashley Schomer, Vesna Bumbasirevic, Bojan Jovanovic, Maja Surbatovic, Milic Veljovic, the ESICM Trials Group, John G. Laffey, Giacomo Bellani, Antonio Pesenti

**Affiliations:** 1 Value-Based Health Care Unit, IRCCS MultiMedica, Sesto San Giovanni, Milan, Italy; 2 Department of Anaesthesia and Intensive Care Medicine, Galway University Hospitals, Galway, Ireland; 3 School of Medicine, Clinical Sciences Institute, National University of Ireland, Galway, Ireland; 4 Department of Medicine and Surgery, University of Milan-Bicocca, Monza, Italy; 5 Department of Emergency and Intensive Care, San Gerardo Hospital, Monza, Italy; 6 Service de Médecine Intensive-Réanimation, Hôpitaux Universitaires Paris-Saclay, Hôpital de Bicêtre, APHP, Le Kremlin-Bicêtre, France; 7 Faculté de Médecine Paris-Saclay, Le Kremlin-Bicêtre, France

**Keywords:** Acute hypoxemic respiratory failure, Acute respiratory distress syndrome, Hospital survival, ICU discharge, LUNG SAFE

## Abstract

**Background:**

To determine the frequency of, and factors associated with, death in hospital following ICU discharge to the ward.

**Methods:**

The Large observational study to UNderstand the Global impact of Severe Acute respiratory FailurE study was an international, multicenter, prospective cohort study of patients with severe respiratory failure, conducted across 459 ICUs from 50 countries globally. This study aimed to understand the frequency and factors associated with death in hospital in patients who survived their ICU stay. We examined outcomes in the subpopulation discharged with no limitations of life sustaining treatments (‘treatment limitations’), and the subpopulations with treatment limitations.

**Results:**

2186 (94%) patients with no treatment limitations discharged from ICU survived, while 142 (6%) died in hospital. 118 (61%) of patients with treatment limitations survived while 77 (39%) patients died in hospital. Patients without treatment limitations that died in hospital after ICU discharge were older, more likely to have COPD, immunocompromise or chronic renal failure, less likely to have trauma as a risk factor for ARDS. Patients that died post ICU discharge were less likely to receive neuromuscular blockade, or to receive any adjunctive measure, and had a higher pre- ICU discharge non-pulmonary SOFA score. A similar pattern was seen in patients with treatment limitations that died in hospital following ICU discharge.

**Conclusions:**

A significant proportion of patients die in hospital following discharge from ICU, with higher mortality in patients with limitations of life-sustaining treatments in place. Non-survivors had higher systemic illness severity scores at ICU discharge than survivors.

*Trial Registration*: ClinicalTrials.gov NCT02010073.

**Supplementary Information:**

The online version contains supplementary material available at 10.1186/s13054-021-03465-0.

## Background

Patients that are discharged alive from the ICU are often considered to have ‘survived’ their critical illness, and to be in the recovery phase. However, this is now understood that these patients suffer ongoing increased morbidity and mortality following the acute phase of their critical illness. Indeed, one might view ICU survival as one—albeit major—of a series of hurdles in a recovery process from critical illness that can take several years. Elegant long-term follow-up studies, such as those conducted by Herridge and colleagues, show substantial ongoing functional limitations that persist up to 5 years following ARDS [1].

In contrast, relatively little is known about the subgroup of patients that are discharged to the ward from the ICU, but subsequently die in hospital prior to discharge. Of particular interest is the identification of potentially modifiable factors associated with in-hospital death in these patients. Patients discharged from ICU can be considered to fall into 2 groups, depending on whether limitations regarding life-sustaining treatments (referred to as ‘treatment limitations’) were placed at the time of discharge [2, 3]. Patients in whom treatment limitations were in place are generally considered to have a more guarded prognosis, while patients without such treatment limitations are thought to have a better prognosis [2, 3].

Given these issues, we wished to examine the frequency and factors associated with death in hospital following ICU discharge in patients enrolled into The Large observational study to UNderstand the Global impact of Severe Acute respiratory FailurE (LUNG SAFE) study, a prospective cohort study undertaken in 459 Intensive Care Units (ICUs) in 50 countries across 5 continents [4]. LUNG SAFE constitutes the largest cohort available of patients with acute hypoxaemic respiratory failure requiring ventilatory support. The wide geographic spread of participating ICUs, and the large patient sample size are important strengths of this study [4]. In this secondary and explorative analysis of LUNG SAFE, our primary objective was to determine the percentage of patients dying in hospital following ICU discharge, in patients with and without treatment limitation decisions in place. Secondary objectives included description of factors associated with death in both patient subgroups, with a particular focus on identifying risk factors (some of which are potentially modifiable) and related to patient management.

## Methods and materials

### Study design

The detailed methods and protocol have been published elsewhere [4]. In brief, LUNG SAFE was an international, multicentre, prospective cohort study, with a 4-week enrolment window in the winter season [4]. The study, funded by the European Society of Intensive Care Medicine (ESICM), was endorsed by multiple national societies/networks (Acknowledgements). All participating ICUs obtained ethics committee approval, and either patient consent or ethics committee waiver of consent. National coordinators (Acknowledgements) and site investigators (Acknowledgements) were responsible for obtaining ethics committee approval and for ensuring data integrity and validity.

### Study population

**Fig. 1:**
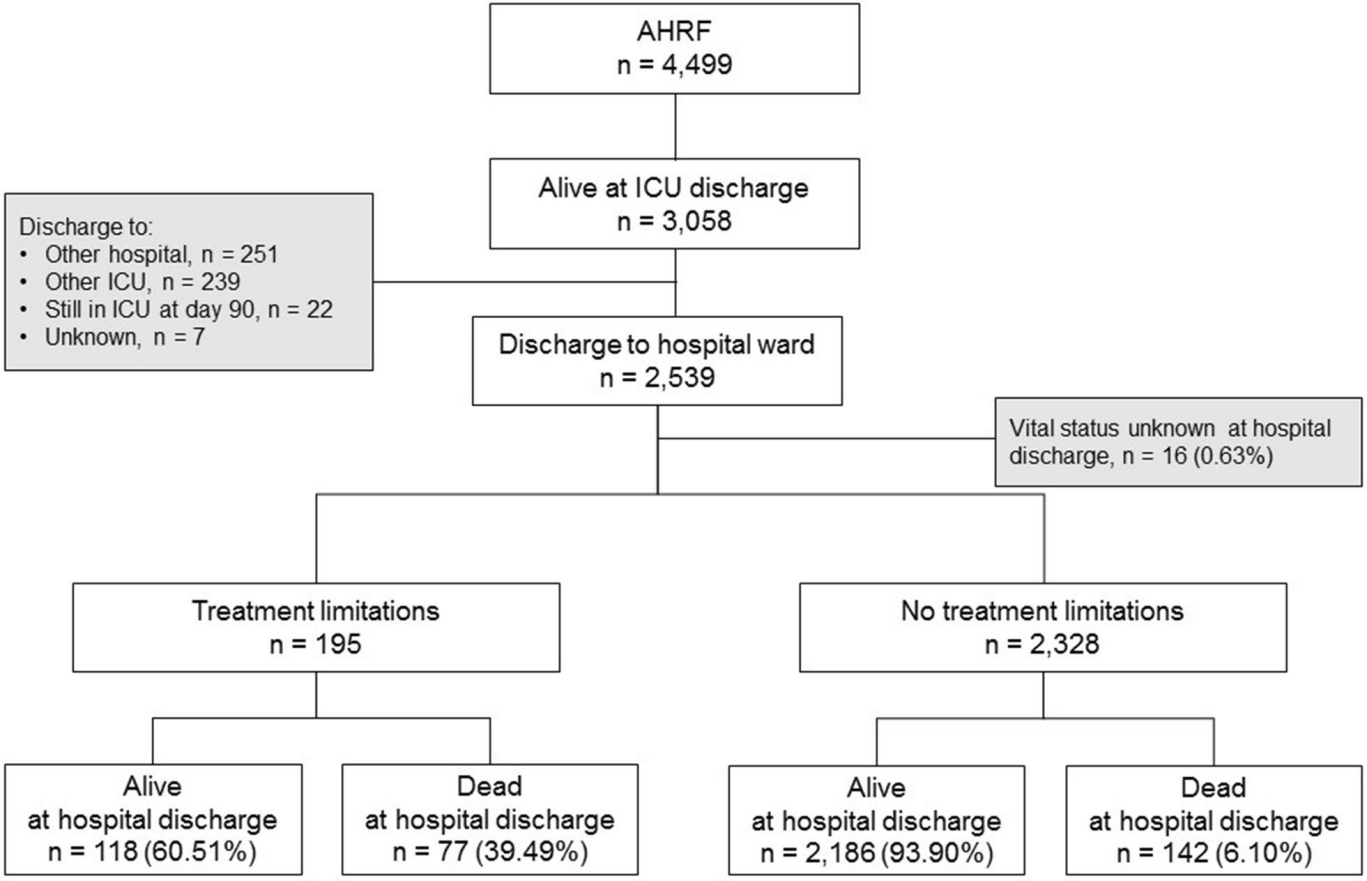
Flowchart of study population subdivided into the patient groups with and without treatment limitations

The study inclusion criteria for acute respiratory hypoxemic failure (AHRF) were: a PaO_2_/FIO_2_ of 300 mmHg or less; new pulmonary infiltrates on chest imaging, and requirement of ventilatory support with a positive end-expiratory pressure (PEEP) of 5 cm H_2_O or more. Exclusion criteria were: age < 16 years or inability to obtain informed consent, where required. The study population consisted of patients fulfilling criteria for AHRF that survived their ICU stay and were discharged to a hospital ward within 90 days of ICU admission). The study population was divided into 2 groups, depending on whether or not the patient has a decision to limit life-sustaining measures (Fig. 1).

### Data definitions

Our data definitions have been previously reported [4]. In the present study, ICU and hospital survival were evaluated at ICU or hospital discharge, or at day 90, whichever occurred first. For the geo-economic area definition, we used the classification we have previously reported [5].

### Data management and statistical analyses

Descriptive statistics included proportions for categorical and mean (standard deviation) or median (interquartile range) for continuous variables. The amount of missing data was low as previously reported [4], and no assumptions were made for missing data. Statistical differences in proportions observed in the groups (treatment limitation, no treatment limitation) were assessed with chi-square test, or Fisher exact test according to number of expected cases. Continuous variables were compared using T-test or Wilcoxon rank sum test, according to Normal data distribution. Shapiro-Wilks test was used to assess normality in data distribution.

In order to assess statistical difference between parameters observed at ICU admission and discharge, we used Wilcoxon signed-rank test accounting for the paired nature of the data not normally distributed.

Logistic regression models were applied in order to identify predictors of hospital mortality in patients without treatment limitation. A stepwise approach was used to detect predictor of hospital mortality after ICU discharge. This approach combines forward and backward selection methods in an iterative procedure (significance level of 0.05 both for entry and retention). Potential independent predictors were: patient characteristics at baseline (age, sex, BMI, geo-economic area), chronic disease (chronic obstructive pulmonary disease (COPD), diabetes mellitus, immuno-incompetence, cardiac failure, renal failure, liver failure), presence of ARDS risk factors, ICU characteristics (number of beds, proportion of ICU beds in hospital, number of beds per physician and per nurse, academic ICU), illness severity parameters evaluated at last available day in ICU (PaO_2_/FiO_2_, PaCO_2_, pH, SOFA score adjusted for missing values). Results were reported as odds ratio with 95% confidence interval. Same approach was used on patients on invasive mechanical ventilation for at least two days during ICU stay in order to assess the possible association between ventilator parameters and hospital mortality (after ICU discharge). The list of possible independent variables used in stepwise approach also included ventilator setting observed during the last available day of IMV. Same analysis was performed on patients with a treatment limitation during ICU stay.

The Kaplan–Meier approach was applied to assess the probability of hospital survival after ICU discharge, considering censored those patients discharged alive from hospital, as well as those patients with a hospital discharge after 60 days from ICU discharge. The log-rank test was used to compare survival curves estimated in patients with or without treatment limitation. Same approach was used to assess probability of hospital survival in study population stratified in 3 groups: patients with a treatment limitation, patients without a treatment limitation and adjunctive measures used during ICU stay, patients without a treatment limitation and no adjunctive measures.

All *p* values were two-sided, with *p* values < 0.05 considered as statistically significant. Statistical analyses were performed with R, version 3.5.2 (The R Foundation for Statistical Computing) and SAS software, version 9.4 (SAS Institute, Cary, NC, USA).

## Results

A total of 4499 patients had AHRF defined by a PaO_2_/FIO_2_ of 300 mmHg or less, new pulmonary infiltrates on chest imaging, and requirement of ventilator support with a PEEP of 5 cm H_2_O or more (Fig. 1). Of these, 3058 (68%) survived to ICU discharge. Of these, 2814 (92%) did not have any treatment limitations in place, while 244 patients (8%) did have limitations in place.

### Death in hospital post-ICU discharge

2186 (94%) of patients without treatment limitations survived to hospital discharge, while 142 (6.1%) died in hospital (Fig. 1). 118 (61%) of patients with treatment limitations survived, while 77 (39%) patients died in hospital. Of the 39 patients (20%) with treatment limitations placed on (36 patients) or before (3 patients) ICU admission, 12 died (32%). Of the 145 patients (74%) that had treatment limitations in place after the day of ICU admission, 62 (43%) died in hospital following ICU discharge. 11 patients had date of limitation not available, of whom 3 died (27%). There were no significant differences in hospital mortality rates (*p* = 0.18).

Patients that died in hospital after ICU discharge differed in a number of potentially important respects from those that survived (Table 1) being older, more likely to have COPD, immunocompromise or chronic renal failure and less likely to have trauma as a risk factor for ARDS.

**Table 1:** Characteristics of study subpopulation with no treatment limitations at ICU discharge according to vital status at hospital discharge.

	Alive *N* = 2186	Dead *N* = 142	Total *N *= 2328	*p *value
Male, n (%)	1363 (62.35)	93 (65.49)	1,456 (62.49)	0.4536
Age (years), mean ± SD	60.03 ± 16.34	68.70 ± 15.71	60.56 ± 16.43	< .0001
Geographic area				0.2086
European high income countries	1207 (55.22)	88 (61.97)	1295 (55.63)	
Non-European high income countries	655 (29.96)	33 (23.24)	688 (29.55)	
Middle income countries	324 (14.82)	21 (14.79)	345 (14.82)	
BMI (kg/m^2^), mean ± SD	27.92 ± 7.72	26.57 ± 6.03	27.84 ± 7.63	0.1389
Length of ICU stay (days) from AHRF onset, median [IQR]	8.00 [5.00–16.00]	10.00 [6.00–16.00]	9.00 [5.00–16.00]	0.1162
Length of ICU stay > 28 days from AHRF onset, *n* (%)	226 (10.34)	8 (5.63)	234 (10.05)	0.0708
Length of ICU stay (days) from admission, median [IQR]	10.00 [5.00–18.00]	10.50 [7.00–18.00]	10.00 [6.00–18.00]	0.0984
ARDS during ICU stay, *n* (%)	1414 (64.68)	93 (65.49)	1507 (64.73)	0.8451
Clinical recognition of ARDS during ICU stay, *n* (%)	925 (42.31)	61 (42.96)	986 (42.35)	0.8806
Chronic disease^a^, *n* (%)				
COPD	492 (22.51)	43 (30.28)	535 (22.98)	0.0328
Diabetes mellitus	501 (22.92)	36 (25.35)	537 (23.07)	0.5047
Immune-incompetence (all-types)	344 (15.74)	40 (28.17)	384 (16.49)	0.0001
Chronic cardiac failure	241 (11.02)	19 (13.38)	260 (11.17)	0.3878
Chronic renal failure	229 (10.48)	23 (16.10)	252 (10.82)	0.0335
Chronic liver failure	42 (1.92)	5 (3.52)	47 (2.02)	0.2054
Risk factors for ARDS, *n* (%)				0.3586
None	373 (17.06)	22 (15.49)	385 (16.97)	
Only non-pulmonary	448 (20.49)	36 (25.35)	484 (20.79)	
Only pulmonary	1110 (50.32)	72 (50.70)	1172 (50.34)	
Both	265 (12.12)	12 (8.45)	277 (11.90)	
Risk factors for ARDS^a^, *n* (%)				
Pneumonia	1089 (49.82)	78 (54.93)	1167 (50.13)	0.2377
Extra-pulmonary sepsis	301 (13.77)	16 (18.31)	327 (14.05)	0.1313
Aspiration of gastric contents	289 (13.22)	13 (9.15)	302 (12.97)	0.1624
Pancreatitis	30 (1.78)	4 (2.82)	43 (1.85)	0.3304
Trauma or pulmonary contusion	152 (6.95)	2 (1.41)	154 (6.62)	0.0100
Inhalation	37 (1.69)	4 (2.82)	41 (1.76)	0.3120
Non cardiogenic shock	124 (5.67)	11 (7.75)	135 (5.80)	0.3055
Drowning	0 (0.00)	0 (0.00)	0 (0.00)	–
Drug overdose	55 (2.52)	3 (2.11)	58 (2.49)	1.0000
Blood transfusion	77 (3.52)	6 (4.23)	83 (3.57)	0.6616
Other risk factors	101 (4.62)	5 (3.52)	106 (4.55)	0.5426
ICU characteristics				
Academic hospital, *n* (%)	1660 (78.01)	106 (75.71)	1766 (77.87)	0.5267
% of ICU on hospital beds, median [IQR]	2.60 [1.56–4.17]	2.61 [1.56–4.35]	2.60 [1.56–4.22]	0.7886
Beds per physician, median [IQR]	4.83 [2.67–10.00]	4.67 [2.67–9.00]	4.83 [2.67–10.00]	0.4145
Beds per nurse, median [IQR]	1.50 [1.00–2.00]	1.31 [1.00–2.00]	1.50 [1.00–2.00]	0.0511

*ARDS* acute respiratory distress syndrome, *BMI* body mass index, *COPD* chronic obstructive pulmonary disease, *ICU* intensive care unit, *IQR* interquartile range [first and third quartile], *SD* standard deviation

^a^Sum of percentages is greater than 100%, because patient could have more than one chronic disease/risk factor.

### Illness severity factors

**Fig. 2:**
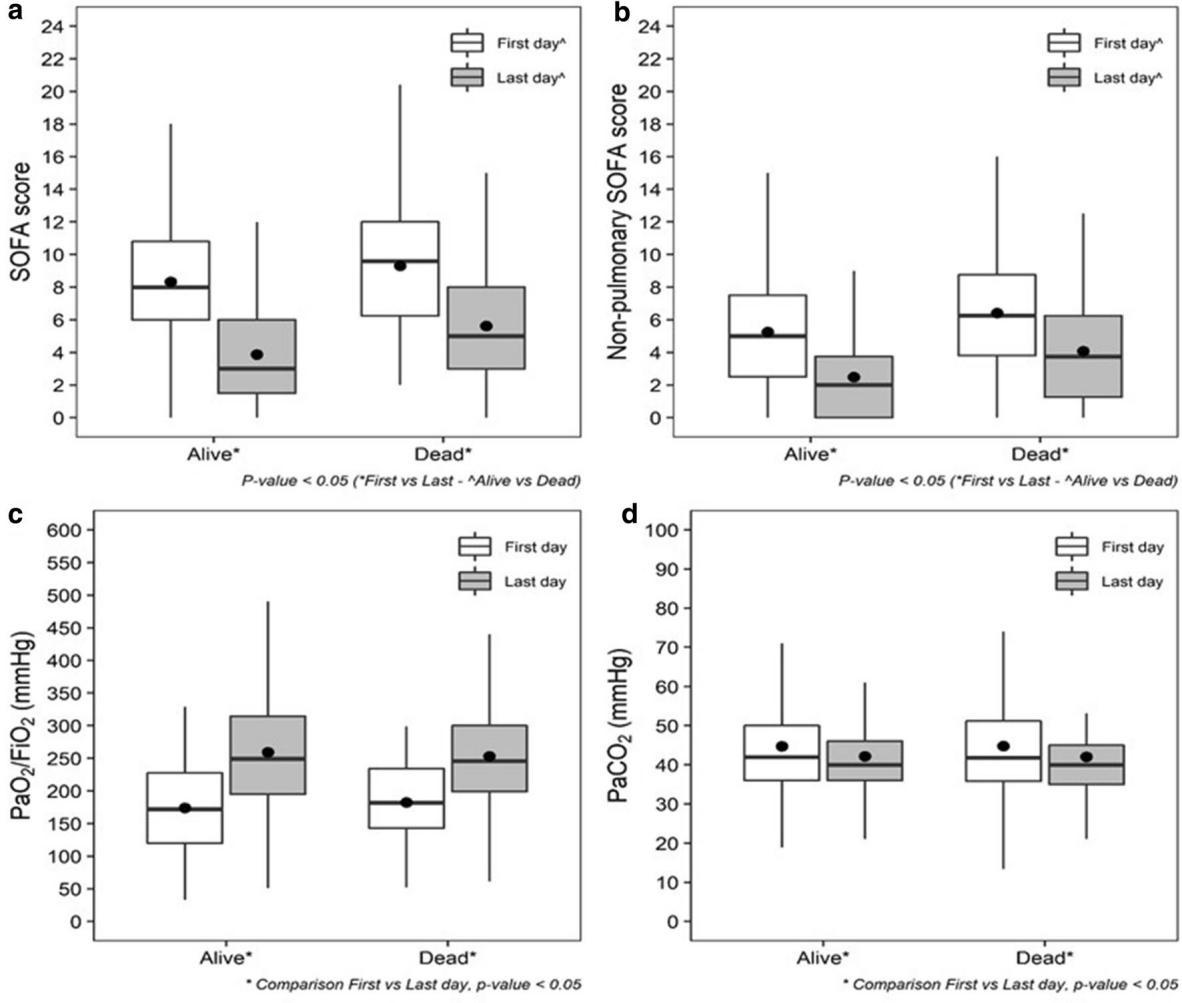
Patients with no treatment limitations who die in hospital following ICU discharge have higher overall SOFA scores (**a**), which appeared to be due to higher systemic organ injury severity scores (**b**) as pulmonary organ injury severity scores (**c**, **d**) were similar, compared to survivors, at both ICU admission and at ICU discharge

Patients with no treatment limitations who died in hospital following ICU discharge had higher organ injury severity scores (Fig. 2a) compared to survivors, at both ICU admission and at ICU discharge (Table 2). SOFA scores at first day of AHRF and at last available day in ICU were higher in patients that died in hospital after ICU discharge (Table 2). This seemed to be driven by the non-pulmonary components of the SOFA score (Fig. 2b), as pulmonary organ injury severity scores did not differ between survivors and non-survivors in those without treatment limitations. Specifically, there was no difference in P/F or PaCO2 on initial or last day between survivors and non-survivors (Fig. 2c, d). In addition, there was no difference in the proportion of patient with ARDS among those that survived versus those that died following ICU discharge (Table 1).

**Table 2:** Illness severity in study subpopulation with no treatment limitations at ICU discharge stratified by vital status at hospital discharge

Parameter	Alive *N* = 2186	Dead *N *= 142	Total *N *= 2328	*p *value
*Illness severity at 1st day of AHRF*				
ARDS, n (%)	1,242 (56.82)	76 (53.52)	1,318 (56.62)	0.4427
Gas exchange				
P_a_O_2_/FiO_2_ (mmHg), mean ± SD	173.81 ± 66.30	182.49 ± 63.28	174.34 ± 66.14	0.1032
SpO_2_ (%), median [IQR]	96.0 [94.0–98.0]	96.0 [94.0–98.0]	96.0 [94.0–98.0]	0.7613
P_a_CO_2_ (mmHg), mean ± SD	45.34 ± 14.88	44.80 ± 14.40	45.31 ± 14.85	0.8351
pH, mean ± SD	7.35 ± 0.10	7.35 ± 0.10	7.35 ± 0.10	0.9699
Adjusted SOFA scores, mean ± SD	8.33 ± 3.72	9.30 ± 3.58	8.39 ± 3.72	0.0010
*Illness severity at last available day in ICU*				
ARDS, *n* (%)	132 (7.65)	8 (7.14)	140 (7.62)	0.8452
Gas exchange				
P_a_O_2_/FiO_2_ (mmHg)				
Mean ± SD	259.00 ± 91.83	252.83 ± 86.61	258.58 ± 91.47	0.7798
Available data, *n* (%)	1163 (53.20)	85 (59.86)	1248 (53.61)	0.1232
SpO_2_ (%)				
Median [IQR]	97.0 [95.0–98.0]	97.0 [95.0–99.0]	97.0 [95.0–98.0]	0.7263
Available data, *n* (%)	1381 (63.17)	89 (62.68)	1470 (63.14)	0.9050
P_a_CO_2_ (mmHg)				
Mean ± SD	42.40 ± 10.97	42.01 ± 11.49	42.38 ± 10.99	0.6008
Available data, *n* (%)	1265 (57.87)	90 (63.38)	1355 (58.20)	0.1969
pH (unit)				
Mean ± SD	7.43 ± 0.05	7.43 ± 0.06	7.43 ± 0.06	0.4129
Available data, *n* (%)	1271 (58.14)	90 (63.38)	1361 (58.46)	0.2197
Adjusted non-pulmonary SOFA scores, mean ± SD				
Mean ± SD	2.49 ± 2.75	4.08 ± 3.37	2.59 ± 2.82	< .0001
Available data, *n* (%)	1337 (61.16)	97 (68.31)	1434 (61.60)	0.0897
Adjusted SOFA scores, mean ± SD				
Mean ± SD	3.87 ± 3.16	5.62 ± 3.71	3.99 ± 3.60	< .0001
Available data, *n* (%)	1341(61.34)	97 (68.31)	1438 (61.77)	0.0979

*ARDS* acute respiratory distress syndrome, *FiO*_*2*_ fraction of inspired oxygen, *IBW* ideal body weight, *ICU* intensive care unit, *IQR* interquartile range [first and third quartile], *P*_*a*_*CO*_*2*_ partial pressure arterial carbon dioxide, *P*_*a*_*O*_*2*_ partial pressure arterial oxygen, *PEEP* positive end-expiratory pressure, *PIP* peak inspiratory pressure, *SD* standard deviation, *SOFA* sequential organ failure assessment.

In contrast, patients with treatment limitations who died in hospital following ICU discharge had comparable systemic organ injury severity scores (Additional file 1: Figure e1A-B), but higher pulmonary organ injury severity scores (Additional file 1: Figure e1C-D) compared to survivors, at both ICU admission and at ICU discharge. ARDS recognition was lower (Additional file 1: Table S1) in non-survivors compared to survivors.

### Patient management factors

**Fig. 3:**
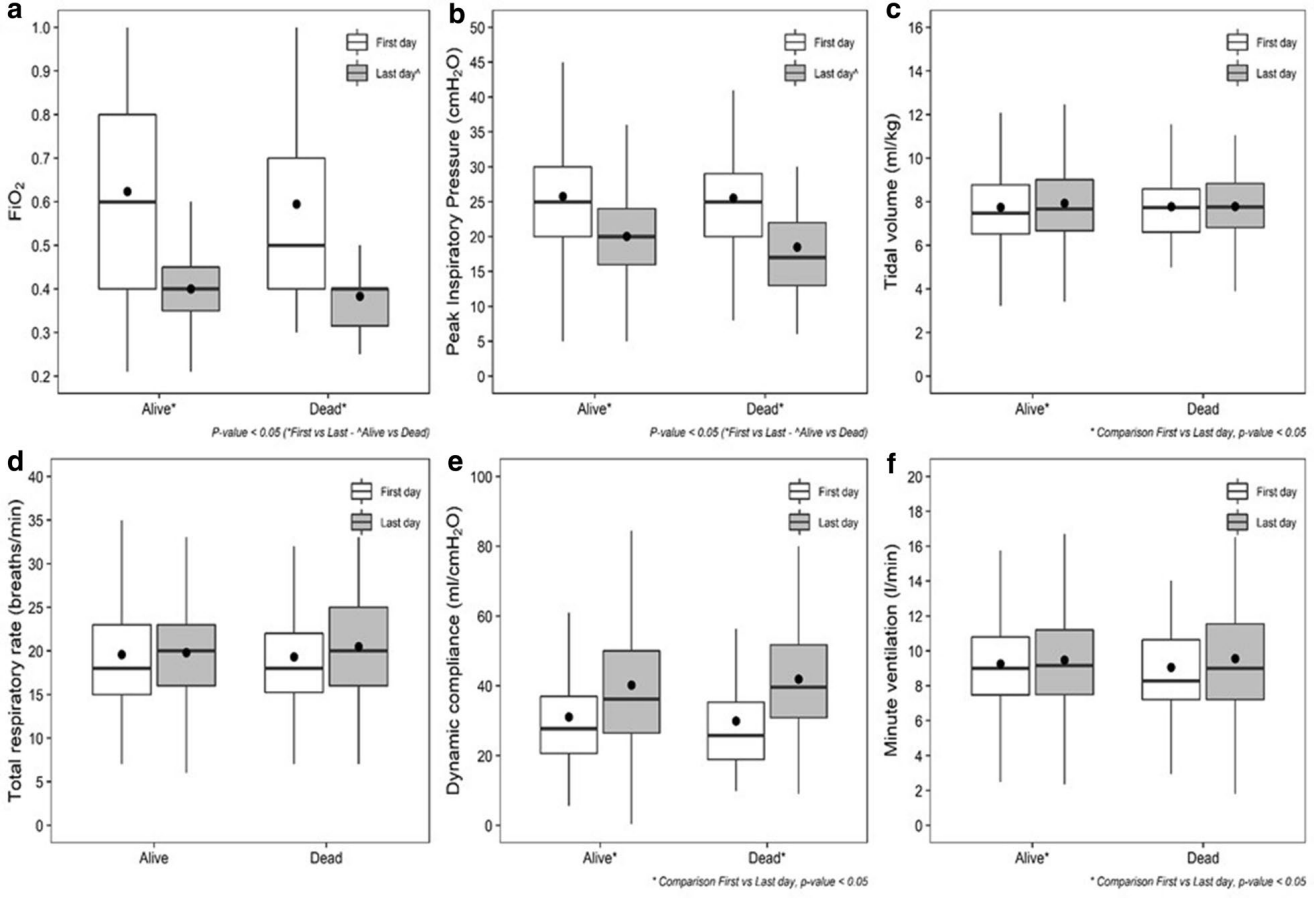
Patients with no treatment limitations who die in hospital require comparable or lower degrees of ventilatory support on the last day of assisted ventilation in the ICU compared to survivors at ICU discharge. Specifically, last day FiO_2_ (**a**) and peak initiatory pressures (**b**) were lower, while tidal volume, respiratory rates, dynamic compliance and minute volumes (**c–f**) were similar, in comparison to hospital survivors

Patients with no treatment limitations that survived to hospital discharge received higher levels of PEEP on the first day of invasive MV (Table 3). In contrast, on the last day of assisted ventilation in the ICU, both surviving and non-surviving patients with no treatment limitations required similar levels of ventilatory support (Table 3). Specifically, last day FiO_2_ (Fig. 3a) and peak initiatory pressures (Fig. 3b) were lower, while tidal volume, respiratory rates, dynamic compliance and minute volumes (Fig. 3c–f) were similar, in comparison to hospital survivors. Furthermore, there were no differences in the length of ICU stay between survivors and non-survivors, although the proportion of patients with long ICU stays was numerically higher in patients that survived post-ICU discharge (Table 1)**.**

**Table 3:** Ventilator setting in patients with no treatment limitations at ICU discharge who received invasive MV for at least 2 days (from AHRF onset) stratified by vital status at hospital discharge

Parameter	Alive *N *= 2186	Dead *N *= 142	Total *N *= 2328	*p* value
Patients on IMV at 1st and 2nd day, *n* (%)	1545 (70.68)	111 (78.17)	1656 (71.13)	0.0562
Last day on IMV (with collected data), median [IQR]	7 [3–20]	7 [3–10]	7 [3–10]	0.7307
Non-invasive Mechanical Ventilation after IMV, *n* (%)	144 (9.32)	7 (6.31)	151 (9.12)	0.2866
Ventilator setting at 1st day of Invasive MV				
Controlled ventilation, *n* (%)	1081 (71.21)	76 (69.72)	1157 (7111)	0.7407
FiO_2_, median [IQR]	0.60 [0.40–0.80]	0.50 [0.40–0.70]	0.57 [0.40–0.80]	0.1899
Set respiratory rate (breaths/min), mean ± SD	17.92 ± 5.83	17.66 ± 5.38	17.90 ± 5.80	0.7580
Total respiratory rate (breaths/min), mean ± SD	19.74 ± 6.14	19.51 ± 5.84	19.73 ± 6.12	0.7573
Tidal volume (ml/kg IBW), mean ± SD	7.77 ± 1.83	7.78 ± 1.88	7.77 ± 1.84	0.6565
High tidal volume (> 8 ml/kg IBW), *n* (%)	543 (36.86)	42 (40.00)	585 (37.07)	0.5203
Dynamic compliance (ml/cmH_2_O), mean ± SD	33.35 ± 23.96	32.23 ± 23.04	33.27 ± 23.89	0.2753
PEEP (cmH_2_O), mean ± SD	8.12 ± 3.21	7.40 ± 2.85	8.07 ± 3.19	0.0095
PIP (cmH_2_O), mean ± SD	26.04 ± 7.99	25.50 ± 8.02	26.01 ± 7.99	0.5395
Plateau pressure measured, n (%)	489 (31.65)	33 (29.73)	522 (31.52)	0.6740
Plateau pressure (cmH_2_O), mean ± SD	21.50 ± 5.62	21.73 ± 6.27	21.52 ± 5.66	0.9205
Driving pressure (cmH_2_O), mean ± SD	13.19 ± 5.03	14.64 ± 5.81	13.28 ± 5.09	0.1699
Minute ventilation (l/min), mean ± SD	9.32 ± 2.87	9.06 ± 3.07	9.30 ± 2.89	0.1545
Standardized minute ventilation (l/min), mean ± SD	10.43 ± 4.45	9.96 ± 3.94	10.40 ± 4.42	0.4408
Ventilator setting at last available day of Invasive MV in ICU				
Controlled ventilation, *n* (%)	384 (25.35)	25 (23.15)	409 (25.20)	0.6112
FiO_2_				
Median [IQR]	0.40 [0.35–0.45]	0.40 [0.30–0.40]	0.40 [0.35–0.45]	0.0309
Available data, *n* (%)	1501 (97.15)	107 (96.40)	1608 (97.10)	0.5586
Total respiratory rate (breaths/min)				
Mean ± SD	20.21 ± 12.15	20.91 ± 6.76	20.26 ± 11.86	0.1920
Available data, *n* (%)	1499 (97.02)	109 (98.20)	1608 (97.10)	0.7674
Tidal volume (ml/kg IBW)				
High tidal volume (> 8 ml/kg IBW), *n* (%)	602 (42.82)	42 (42.00)	644 (42.76)	0.8733
Mean ± SD	7.95 ± 2.06	7.89 ± 2.04	7.95 ± 2.06	0.9763
Available data, *n* (%)	1406 (91.00)	100 (90.09)	1506 (90.94)	0.7461
Dynamic compliance (ml/cmH_2_O)				
Mean ± SD	49.39 ± 45.70	48.72 ± 30.39	49.35 ± 44.84	0.1701
Available data, *n* (%)	1344 (86.99)	96 (86.49)	1440 (86.96)	0.8790
PEEP (cmH_2_O)				
Mean ± SD	6.66 ± 2.43	6.26 ± 1.95	6.63 ± 2.40	0.0597
Available data, *n* (%)	1496 (96.83)	106 (95.50)	1602 (96.74)	0.4043
PIP (cmH_2_O)				
Mean ± SD	20.17 ± 7.16	18.51 ± 7.01	20.06 ± 7.16	0.0079
Available data, *n* (%)	1395 (90.29)	99 (89.19)	1494 (90.22)	0.7058
Minute ventilation (l/min)				
Mean ± SD	9.65 ± 4.08	9.56 ± 3.25	9.64 ± 4.03	0.9026
Available data, *n* (%)	1447 (93.66)	103 (92.79)	1550 (93.60)	0.7194
Standardized minute ventilation (l/min)				
Mean ± SD	10.11 ± 4.93	10.04 ± 4.00	10.10 ± 4.87	0.7355
Available data, *n* (%)	1257 (81.36)	92 (82.88)	1349 (81.46)	0.6899

*FiO*_*2*_ fraction of inspired oxygen, *IBW* ideal body weight, *IMV* invasive mechanical ventilation, *IQR* interquartile range [first and third quartile], *PEEP* positive end-expiratory pressure, *PIP* peak inspiratory pressure, *SD* standard deviation

Similar patterns were seen in patients with treatment limitations who died in hospital (Additional file 1: Figure e2A-F). Patients with treatment limitations who died post ICU discharge had shorter ICU stays compared to those that survived (Additional file 1: Table S1).

### Impact of adjunctive therapies

The frequency of neuromuscular blockade use, and of any adjunctive treatment was reduced in patients who died in hospital following ICU discharge (Table 4). Use adjunctive measures was independently associated with reduced hospital mortality in a multivariate logistic regression model (Table 5).

**Table 4:** Adjunctive measures performed during ICU stay in study subpopulation with no treatment limitations at ICU discharge stratified by vital status at hospital discharge

Parameter	Alive *N *= 2186	Dead *N *= 142	Total *N *= 2328	*p* value
Neuromuscular blockade, *n* (%)	304 (13.91)	8 (5.63)	312 (13.40)	0.0050
Recruitment maneuvers, *n* (%)	305 (13.95)	14 (9.86)	319 (13.70)	0.1693
Prone positioning, *n* (%)	100 (4.57)	2 (1.41)	102 (4.38)	0.0741
ECMO, *n* (%)	30 (1.37)	0 (0.00)	30 (1.29)	0.1600
Inhaled vasodilators, *n* (%)	126 (5.76)	7 (4.93)	133 (5.71)	0.6781
HFOV, *n* (%)	27 (1.24)	0 (0.00)	27 (1.16)	0.1828
None of above adjunctive measures, *n* (%)	1571 (71.87)	116 (81.69)	1687 (72.47)	0.0111

*ECMO* extra corporeal membrane oxygenation, *HFOV* high frequency oscillatory ventilation

**Table 5:** SOFA Scores and Outcomes in patients with no treatment limitations by major Geo-Economic Area

Parameter	Alive *N *= 2186	Dead *N *= 142	Total *N *= 2328	*p *value
*Illness severity at last available day in ICU*				
Adjusted non-pulmonary SOFA scores, median [1st–3rd quartile]				
European high income countries (*n *= 1295)	1.25 [0.00–3.00]	3.75 [1.25–5.00]	1.25 [0.00–3.75]	< .0001
Non-European high income countries (*n *= 688)	3.00 [1.00–5.00]*	7.00 [3.75–9.00]	3.00 [1.00–5.00]	0.0002
Middle income countries (*n *= 345)	1.67 [0.00–4.00]°	2.00 [0.00–5.00]	1.67 [0.00–4.00]	0.6691
*p* value (comparison among areas)	< .0001	0.0046	< .0001	
*Illness severity on Day 10 in ICU*				
Adjusted non-pulmonary SOFA scores, median [1st–3rd quartile]				
European high income countries (*n *= 1295)	1.67 [0.00–3.00]	4.0 [2.5–5.00]	1.67 [0.00–3.75]	0.0076
Non-European high income countries (*n *= 688)	2.00 [0.00–5.00]*	5.63 [3.75–9.00]	2.75 [0.00–5.00]	0.0160
Middle income countries (*n *= 345)	2.00 [0.00–4.00]°	2.50 [0.00–6.50]	2.00 [0.00–5.00]	0.8256
*p* value (comparison among areas)	0.2436	0.3817	0.1790	

*p value < 0.05, comparison with European high-income countries

°*p* value < 0.05, comparison with Non-European high-income countries

### Impact of geo-economic area

Patients from the Non-European-high income area with no treatment limitations had higher SOFA scores at ICU discharge than patients from Europe or from Middle Income countries (Table 5). However, SOFA scores at day 10 post ICU admission were not different across the regions. In patients without treatment limitations, ward mortality ratios were not different (*p* = 0.2086) between geographic areas (Table 1). In patients with treatment limitations of LSMs, ward mortality ratios were significantly lower in middle income countries (17.2%) compared to both European-High (41.5%; *p* = 0.0160) and Non-European-High (46.7%, *p* = 0.0071) income countries (Table 1 and Additional file 1: Table S1).

### Factors associated with hospital death post ICU discharge

**Fig. 4:**
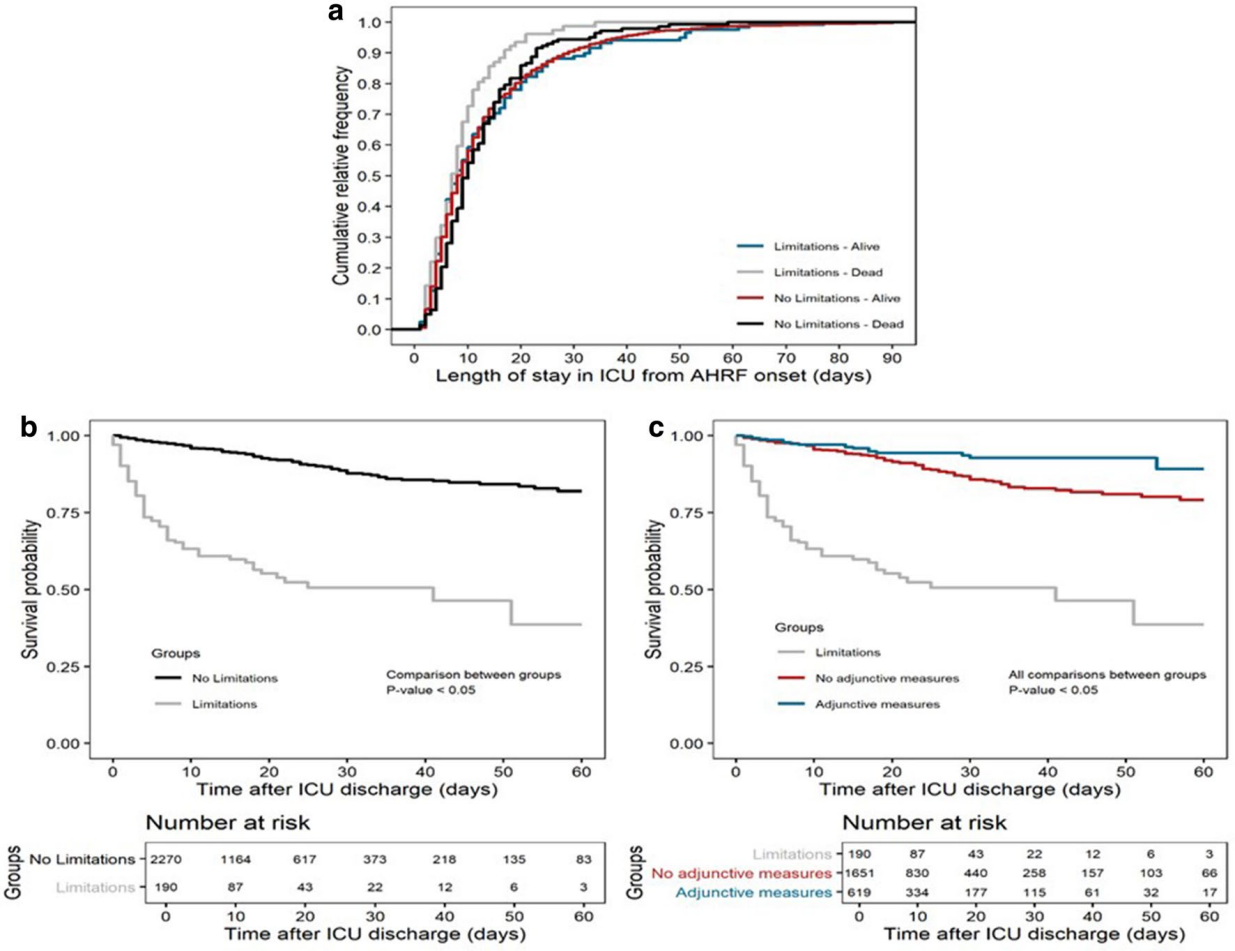
Outcomes of patients that survive to hospital discharge. In **a** length of ICU stay was similar in patients who survived to hospital discharge and those that died, both with and without treatment limitations. In **b,** hospital survival rates post ICU discharge were significantly lower in patients that had treatment limitations, compared to those with no limitations. In **c** in patients with no limitations, survival was significantly higher in those that received adjunctive therapies

Length of ICU stay was similar in patients who survived to hospital discharge and those that died, in patients without treatment limitations (Fig. 4a). In contrast, patients with treatment limitations who died post ICU discharge had shorter ICU stays compared to those that survived (Additional file 1: Table S1). Hospital survival rates post ICU discharge were significantly lower in patients that had treatment limitations, compared to those with no limitations (Fig. 4b). In patients without treatment limitations, there were more deaths following ICU discharge in patients who received no adjunctive treatment as part of their ARDS management (Fig. 4c). The majority deaths for patients with limitations in life-sustaining therapies occurred within 10 days of discharge from the ICU (Fig. 4b).

In a multivariate logistic regression model Factors associated with increased hospital mortality in patients with no limitations of life sustaining measures included age, adjusted SOFA score on the last available day in ICU, and immune-incompetence. Duration of ICU stay was also associated with hospital mortality post ICU discharge in patients that received at least 2 days of invasive mechanical ventilation (Table 6). Factors associated with hospital mortality in patients with treatment limitations at ICU discharge are presented in Additional file 1: Table S5.

**Table 6:** Factors associated with hospital mortality in patients with no treatment limitations at ICU discharge

	OR (95% CI)	*p* value
Multivariable logistic regression model 1 (*n* = 1438 on 2328)		
Age (years)	1.044 (1.028–1.061)	< .0001
Adjusted SOFA score at last available day in ICU	1.154 (1.090–1.222)	< .0001
Immune-incompetence (ref. No)	2.086 (1.281–3.397)	0.0031
Adjunctive measures during ICU stay (ref. No)	0.574 (0.340–0.970)	0.0383
Multivariable logistic regression model on patients on MV for at least 2 days (from AHRF onset) (*n *= 1043 on 1656)		
Age (years)	1.048 (1.029–1.067)	< .0001
Adjusted SOFA score at last available day in ICU	1.167 (1.091–1.248)	< .0001
Immune-incompetence (ref. No)	1.938 (1.071–3.509)	0.0288
BMI (kg/m^2^)	0.954 (0.911–0.999)	0.0469

Model 1 was identified by stepwise approach using as possible predictors: baseline patients’ characteristics, parameters of illness of severity at last available day in ICU, use of adjunctive measures during ICU stay and ICU characteristics.

Model 2 was identified by stepwise approach using as possible predictors: baseline patients’ characteristics, parameters of illness of severity and ventilator setting at last available day in ICU, use of adjunctive measures during ICU stayand ICU characteristics

*CI* confidence interval, *ICU* intensive care unit, *OR* odds ratio, *SOFA* sequential organ failure assessment

## Discussion

In the current study, we found that 94% of patients without limitations of life sustaining therapy that were discharged from ICU stay survived to hospital discharge, while 61% of patients who had treatment limitations in place also survived to hospital discharge. Patients without treatment limitations that died in hospital after ICU discharge were older, and more likely to have COPD, immunocompromise or chronic renal failure. They were less likely to have trauma as a risk factor for ARDS, or to receive neuromuscular blockade or any adjunctive measure. An important—and unexpected—finding was that even though these patients were critically ill due to ARDS, it was the non-pulmonary components of their organ dysfunction that was associated with risk of death in hospital following ICU discharge, while the derangement of oxygenation at ICU admission was not. In addition, our finding that non-survivors received less adjunctive therapies than survivors, raises important questions on whether the low implementation of these measures might contribute to some of the long-term ARDS mortality. Understanding the factors associated with death in hospital following ICU discharge may allow us to focus efforts on these issues in order to improve outcomes.

### Factors contributing to death post ICU discharge

Our finding of a 6% mortality post ICU discharge in patients with acute hypoxaemic respiratory failure is at the lower end of a range of 6–25% hospital mortality rates reported in ICU survivors in earlier studies [6–8]. However, it remains higher than other studies of ICU survivors where it has ranged from 3% in patients at risk for ARDS to 4% in all ICU patients without limitations in life sustaining therapies [9, 10]. In this regard it is important to remember that the LUNG SAFE population constitutes a more severely ill patient cohort, with patients all fulfilling criteria for severe hypoxaemia requiring assisted ventilation.

Identifying risk factors in patients who are likely to die in hospital following ICU discharge may allow us to focus efforts on these factors (if modifiable) in order to improve outcomes, either prior to or following ICU discharge. In this study, we found that patients dying post ICU discharge were systemically sicker as indicated by non-pulmonary SOFA at ICU discharge. Sepsis is a frequent cause of later deaths in patients with ARDS [11], which may be consistent with our finding of a higher non-pulmonary SOFA score for patients that died post ICU discharge. In contrast, pulmonary factors, including initial ARDS severity or respiratory status at weaning from invasive ventilation, were not associated with hospital mortality post ICU discharge.

In regard to patient management, patients that received either neuromuscular blockade use or the use of any adjunct, were more likely to survive post ICU discharge. However, this finding needs to be balanced against our prior findings showing that ICU survival in patient receiving adjunctive therapies was lower [12], raising the potential that this finding may reflect an alteration in the pattern of patients dying in the ICU versus the wards, rather than a true association with improved patient outcome.

The duration of ICU stay was similar in patients that survived following ICU discharge compared to those that died in-hospital, with the proportion of longer ICU stay patients significantly higher in survivors. In patients with treatment limitations, non-survivors actually had shorter ICU stays compared to survivors. This finding appears to rule out the potential for patients that die post ICU discharge to have had longer durations of critical illness compared to patients that survive to hospital discharge.

#### Impact of limitation of care

Our findings suggest that the likelihood of hospital survival post-ICU discharge varies greatly depending on whether or not treatment limitations are in place. This finding is consistent with previous studies showing that the presence of limitations of life sustaining therapies is the most important factor in predicting death post ICU admission [2].

The majority of decisions to limit of life sustaining therapies were made after development of AHRF. Interestingly, hospital survival post ICU discharge in patient with a treatment limitation decision was encouragingly high at 61%, while the timing of placement of treatment limitations didn’t significantly affect the mortality rate.

Increased age and the presence of active or hematologic neoplasm, immune suppression, chronic liver failure and indices of greater illness severity were associated with limitation of care, consistent with prior findings [10]. Overall, there were similarities between the factors associated with patient outcome, and those associated with limitation of care. This may be consistent with the fact that death in the ICU frequently occurs in the context of decisions to limit life sustaining therapy due to perceived futility [13, 14]. Of interest ARDS recognition rates in patients with limitations of care were lower in non-survivors compared to survivors, a finding not seen in patients with no treatment limitations.

Encouragingly, the majority of patients with treatment limitations survived their hospital stay in this cohort. This finding is consistent with reports of improved outcomes for patients with limitations of life sustaining therapies in other recent studies. In a prospective observational study of 22 European ICUs, significantly more patients had limitations in life-sustaining therapies, while death without limitations in life-sustaining therapies occurred significantly less frequently, in 2015–2016 compared with 1999–2000 [3]. Consistent with our findings, overall survival in patients with treatment limitations was better in the 2015–6 cohort (20.4%) compared to the 1999–2000 cohort (5.5%). Our findings further suggest that, in the patient cohort with treatment limitations that survive to ICU discharge, the chances of survival to hospital discharge are quite favourable.

#### Geo-economic area

The higher SOFA sores at ICU discharge in patients from the Non-European-high income area may be explained by their shorter ICU stays [5], given that SOFA scores at day 10 post ICU admission were not different across the regions. Geo-economic location was not an independent predictor of survival in hospital post ICU discharge. The substantially higher proportion of survival patients with treatment limitations in Middle Income countries suggests these patients may be different to patients with limitations in high income countries, possibly because limitations were placed for reasons other than anticipated poor prognosis.

#### Study limitations

There are limitations to this study. Our study focused on identifying factors during the ICU stay, and did not examine factors following ICU discharge, that were associated with death in this population. It would likely have yielded further insights into this important area if we had collected more detailed data on the status of patients post ICU discharge, such as the Sabadell score [15]. We do not have data on any decisions made regarding limitation of life supporting measures following ICU discharge. However, our aim was to look at factors relating to the ICU stay and their impact on mortality post discharge, as this is the aspect of care under the control of the ICU team. This is not to negate the impact of events following ICU discharge on patient outcomes. In this regard, recent findings that adverse events occur commonly following ICU discharge, and can contribute to death in hospital are of particular relevance [16].

We do not have data on whether care-providers instituted treatment limitations in some patients once discharged from the ICU, nor do we have information on where patients were discharged to such as home or nursing home. We did not record details of the specific life supporting measures that were limited, or whether there were more than one decision made regarding these measures during the ICU stay.

This is a secondary and exploratory analysis of LUNG SAFE observational study and no prespecified hypotheses on mortality risk after ICU discharge were considered during study conception. As this was an exploratory study, no adjustments were made to significance levels for multiple comparison testing.

As this is an observational study, we cannot ascribe causation to factors that were associated with better outcomes including adjunctive measure use. Similar to other epidemiologic studies, we did not have access to the source data for the patients in the enrolling ICUs, and it is possible that some patients with hypoxemia, and thus ARDS, in participating centres were missed. It is important to stress, however, that ICUs were participating whether or not they identified any patient having ARDS and that the diagnosis of ARDS was not based on chart records. In addition, enrolment of patients with ARDS from participating ICUs met expectations based on their recorded 2013 admission rates, while data from lower recruiting ICUs were not different from higher enrolling ICUs, suggesting the absence of reporting biases. To ensure data quality, we instituted a robust data quality control program in which all centres were requested to verify data that appeared inconsistent or erroneous. The absence of data on other aspects of ICU management, e.g. fluid therapy, may limit the conclusions that can be drawn.

## Conclusions

This is the first study to our knowledge examining factors associated with mortality post ICU discharge in patients with ARDS. Encouragingly, survival rates to hospital discharge following ICU discharge are high, with survival rates in patients with limitations of life sustaining therapy higher than expected. An important—and unexpected—finding was that even though these patients were critically ill due to ARDS, it was the non-pulmonary components of their organ dysfunction that was associated with risk of death in hospital following ICU discharge. In addition, our finding that non-survivors received less adjunctive therapies than survivors, raises important questions.

Focusing attention on this and other factors associated with death in hospital following ICU discharge may allow us to further improve outcomes in these patients.

## Supplementary Information

**Additional file 1.** Supplemental Results.

## Data Availability

The data that support the findings of this study were made available by the European Society of Intensive Care Medicine. Restrictions apply to the availability of these data, which were used after approval was granted by the executive committee for the OPEN-LUNG SAFE initiative. Further details about accessing these data can be found online (https://www.esicm.org/research/trials/trials-group-2/lung-safe/).

## Abbreviations

LUNG SAFE: Large observational study to UNderstand the Global impact of Severe Acute respiratory FailurE
ICUs: Intensive Care Units
ESICM: European Society of Intensive Care Medicine
AHRF: Acute hypoxemic respiratory failure
PEEP: Positive end-expiratory pressure
