# Supplementary material for: Death in hospital following ICU discharge: insights from the LUNG SAFE study
Source: Crit Care. 2021 Apr 13;25:144. doi: 10.1186/s13054-021-03465-0 (PMC8043098; doi:10.1186/s13054-021-03465-0)
Supplement: Supplementary file 1 — Additional file 1. Supplemental Results. [file 13054_2021_3465_MOESM1_ESM.docx]

**Title:** Death in hospital following ICU discharge: insights from the LUNG SAFE study

**Authors:** Madotto F*, McNicholas B*, Rezoagli E, Pham T, Laffey JG^¥^, Bellani G, On behalf of the LUNG SAFE Investigators and the ESICM Trials Group.

**SUPPLEMENTAL RESULTS**

***Figure e1***: Patients with treatment limitations who die in hospital following ICU discharge have similar systemic organ injury severity scores (***Panels A and B***) but higher pulmonary organ injury severity scores (***Panels C and D***) compared to survivors, at both ICU admission and at ICU discharge.

**
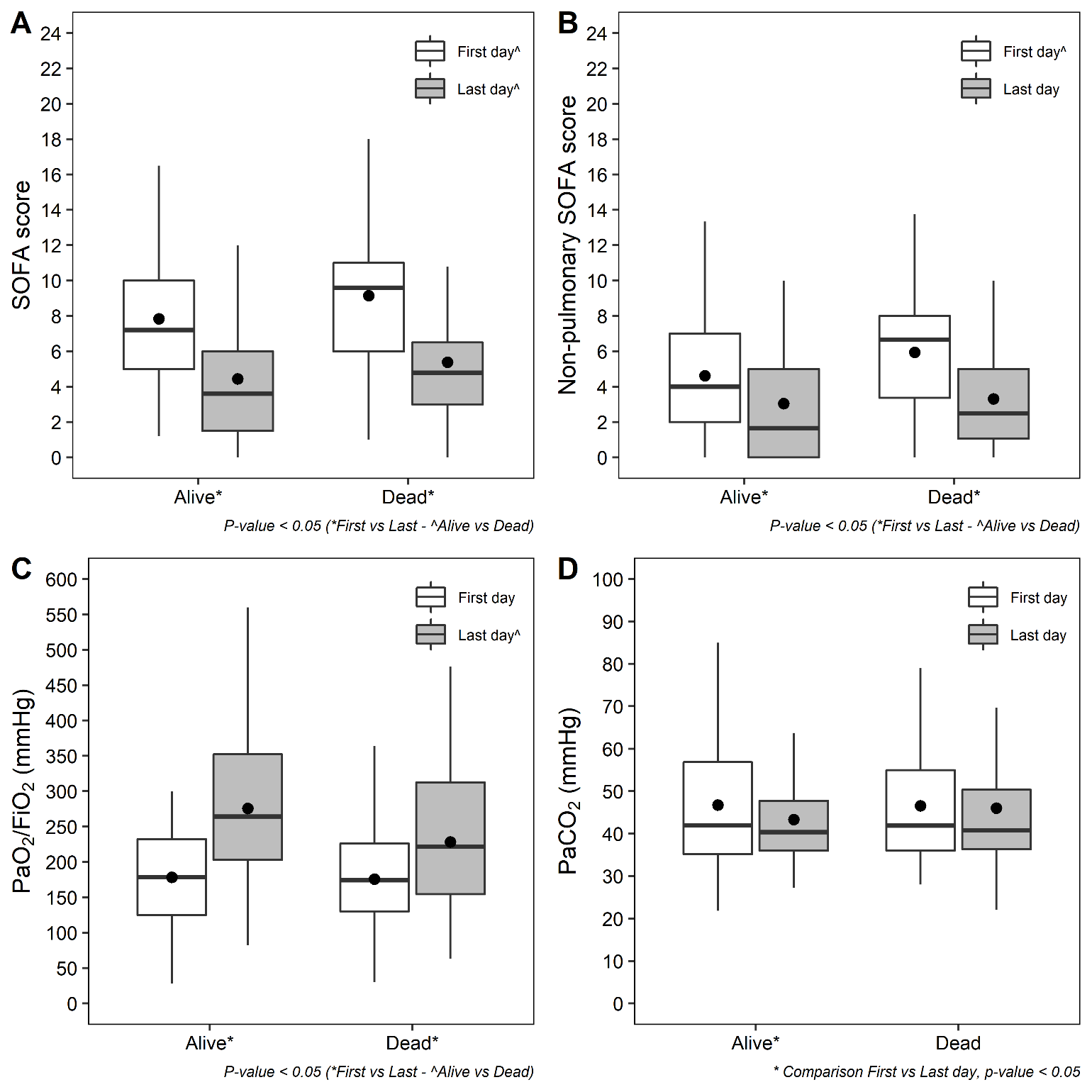
**

***Figure e2*** – Patients with treatment limitations who die in hospital require comparable or lower degrees of ventilatory support on the last day of assisted ventilation in the ICU compared to survivors at ICU discharge.

**
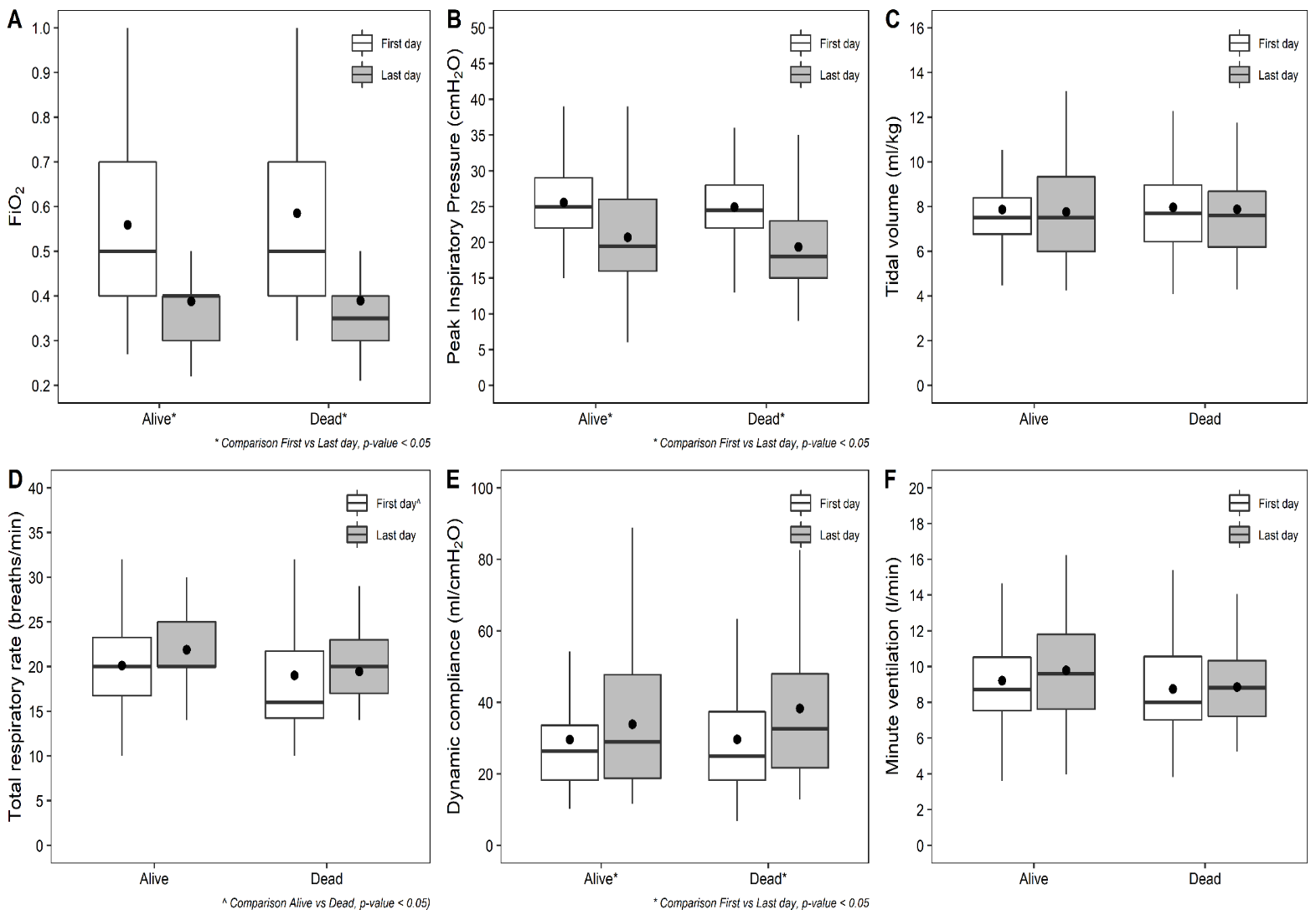
**

**Table S1.** Characteristics of patients with treatment limitations at ICU discharge according to vital status at hospital discharge.

|  | **Alive**  **N=118** | **Dead**  **N=77** | **Total**  **N=195** | *p-value* |
| --- | --- | --- | --- | --- |
| Male, n (%) | 65 (55.08) | 45 (58.44) | 110 (56.41) | *0.6440* |
| **Age (years), mean ± SD** | 69.05 ± 17.26 | 72.82 ± 14.04 | 70.54 ± 16.13 | *0.2065* |
| Geographic area |  |  |  | **0.0237** |
| European high income countries | 62 (52.54) | 44 (57.14) | 106 (54.36) | *0.5284* |
| Non-European high income countries | 32 (27.12) | 28 (36.36) | 60 (30.77) | *0.1715* |
| Middle income countries | 24 (20.34) | 5 (6.49) | 29 (14.87) | *0.0079* |
| BMI (kg/m^2^), mean ± SD | 25.71 ± 6.43 | 25.74 ± 5.98 | 25.72 ± 6.24 | *0.7156* |
| Length of ICU stay (days) from AHRF onset, median [IQR] | 8.0 [5.0-17.0] | 7.0 [4.0-11.0] | 8.0 [4.0-14.0] | *0.0723* |
| Length of ICU stay > 28 days from AHRF onset, n (%) | 14 (11.86) | 1 (1.30) | 15 (7.69) | ***0.0068*** |
| Length of ICU stay (days) from admission, median [IQR] | 9.0 [5.0-18.0] | 8.0 [5.0-11.0] | 8.0 [5.0-15.0] | ***0.0372*** |
| ARDS during ICU stay, n (%) | 85 (72.03) | 52 (67.53) | 137 (70.26) | *0.5015* |
| Clinical recognition of ARDS during ICU stay, n (%) | 50 (42.37) | 19 (24.68) | 69 (35.38) | *0.0115* |
| Chronic disease^§^, n (%) |  |  |  |  |
| COPD | 33 (27.97) | 21 (27.27) | 54 (27.69) | *0.9158* |
| Diabetes mellitus | 32 (27.12) | 25 (32.47) | 57 (29.23) | *0.4221* |
| Immune-incompetence (all-types) | 22 (18.64) | 17 (22.08) | 39 (20.00) | *0.5579* |
| Chronic cardiac failure | 22 (18.64) | 10 (12.99) | 32 (16.41) | *0.2971* |
| Chronic renal failure | 11 (9.32) | 14 (18.18) | 25 (12.82) | *0.0705* |
| Chronic liver failure | 0 (0.00) | 3 (3.90) | 3 (1.54) | *0.0601* |
| Risk factors for ARDS, n (%) |  |  |  | *0.4840* |
| None | 17 (14.41) | 16 (20.78) | 33 (16.92) |  |
| Only non-pulmonary | 21 (17.80) | 14 (18.18) | 35 (17.95) |  |
| Only pulmonary | 70 (59.32) | 38 (49.35) | 108 (55.38) |  |
| Both | 10 (8.47) | 9 (11.69) | 19 (9.74) |  |
| Risk factors for ARDS^§^, n (%) |  |  |  |  |
| Pneumonia | 65 (55.08) | 40 (51.95) | 105 (53.85) | *0.6676* |
| Extra-pulmonary sepsis | 13 (11.02) | 13 (16.88) | 26 (13.33) | *0.2388* |
| Aspiration of gastric contents | 17 (14.41) | 8 (10.39) | 25 (12.82) | *0.4121* |
| Pancreatitis | 0 (0.00) | 0 (0.00) | 0 (0.00) | *-* |
| Trauma or pulmonary contusion | 7 (5.93) | 1 (1.30) | 8 (4.10) | *0.1502* |
| Inhalation | 7 (5.93) | 1 (1.30) | 8 (4.10) | *0.1502* |
| Non cardiogenic shock | 4 (3.39) | 5 (6.49) | 9 (4.62) | *0.3214* |
| Drowning | 0 (0.00) | 0 (0.00) | 0 (0.00) | *-* |
| Drug overdose | 1 (0.85) | 0 (0.00) | 1 (0.51) | *1.0000* |
| Blood transfusion | 3 (2.54) | 4 (5.19) | 7 (3.59) | *0.4374* |
| Other risk factors | 7 (5.93) | 5 (6.49) | 12 (6.15) | *1.0000* |
| ICU characteristics |  |  |  |  |
| Academic hospital, n (%) | 78 (70.27) | 58 (76.32) | 136 (72.73) | *0.3619* |
| % of ICU on hospital beds, median [IQR] | 2.40 [1.40-3.16] | 2.50 [1.37-3.91] | 2.40 [1.40-3.33] | *0.3163* |
| Beds per physician, median [IQR] | 4.50 [3.00-8.00] | 5.00 [3.00-10.00] | 4.50 [3.00-8.00] | *0.1803* |
| Beds per nurse, median [IQR] | 1.50 [1.00-2.29] | 1.55 [1.06-2.00] | 1.50 [1.00-2.10] | *0.9380* |

*Abbreviations: ARDS: acute respiratory distress syndrome; BMI: body mass index; COPD: chronic obstructive pulmonary disease; ICU: intensive care unit; IQR: interquartile range [first and third quartile]; SD: standard deviation.*

*§ Sum of percentages is greater than 100%, because patient could have more than one chronic disease/risk factor.*

**Table S2.** Illness severity in patients with treatment limitations at ICU discharge stratified by vital status at hospital discharge.

| **Parameter** | **Alive**  **N=118** | **Dead**  **N=77** | **Total**  **N=195** | *p-value* |
| --- | --- | --- | --- | --- |
| **Illness severity at 1^st^ day of AHRF** |  |  |  |  |
| ARDS, n (%) | 77 (65.25) | 46 (59.74) | 123 (63.08) | *0.4354* |
| Gas exchange |  |  |  |  |
| P_a_O_2_/FiO_2_ (mmHg), mean ± SD | 178.37 ± 71.45 | 175.75 ± 62.13 | 177.32 ± 67.73 | *0.7937* |
| SpO_2_ (%), median [IQR] | 96.0 [94.0-98.0] | 96.0 [94.0-98.0] | 96.0 [94.0-98.0] | *0.7295* |
| P_a_CO_2_ (mmHg), mean ± SD | 47.32 ± 16.29 | 46.54 ± 15.50 | 47.01 ± 15.95 | *0.7885* |
| pH, mean ± SD | 7.34 ± 0.11 | 7.36 ± 0.11 | 7.35 ± 0.11 | *0.3171* |
| Adjusted SOFA scores, mean ± SD | 7.84 ± 3.89 | 9.14 ± 3.97 | 8.35 ± 3.97 | ***0.0044*** |
| **Illness severity at last available day in ICU** |  |  |  |  |
| ARDS, n (%) | 7 (8.14) | 12 (19.05) | 19 (12.75) | ***0.0486*** |
| Gas exchange |  |  |  |  |
| P_a_O_2_/FiO_2_ (mmHg) |  |  |  |  |
| Mean ± SD | 275.73 ± 106.31 | 228.15 ± 97.19 | 256.86 ± 104.99 | ***0.0163*** |
| Available data, n (%) | 70 (59.32) | 46 (59.74) | 116 (59.49) | *0.9536* |
| SpO_2_ (%) |  |  |  |  |
| Median [IQR] | 97.0 [94.0-98.0] | 96.0 [95.0-99.0] | 96.5 [94.0-98.0] | *0.5405* |
| Available data, n (%) | 76 (64.41) | 50 (64.94) | 126 (64.62) | *0.9399* |
| P_a_CO_2_ (mmHg) |  |  |  |  |
| Mean ± SD | 43.29 ± 11.26 | 46.03 ± 16.90 | 44.34 ± 13.70 | *0.6560* |
| Available data, n (%) | 74 (62.71) | 46 (59.74) | 120 (61.54) | *0.6767* |
| pH (unit) |  |  |  |  |
| Mean ± SD | 7.41 ± 0.07 | 7.42 ± 0.07 | 7.41 ± 0.07 | *0.2578* |
| Available data, n (%) | 74 (62.71) | 47 (61.04) | 121 (62.05) | *0.8140* |
| Adjusted SOFA scores, mean ± SD |  |  |  |  |
| Mean ± SD | 4.44 ± 3.85 | 5.38 ± 3.18 | 4.84 ± 3.60 | ***0.0334*** |
| Available data, n (%) | 69 (58.47) | 51 (66.23) | 120 (61.54) | *0.2763* |
| Adjusted non-pulmonary SOFA scores, mean ± SD |  |  |  |  |
| Mean ± SD | 3.04 ± 3.41 | 3.31 ± 2.93 | 3.16 ± 3.21 | ***0.2738*** |
| Available data, n (%) | 69 (58.47) | 50 (64.94) | 119 (61.03) | *0.3659* |

*Abbreviations: ARDS: acute respiratory distress syndrome; FiO_2_: fraction of inspired oxygen; IBW: ideal body weight; ICU: intensive care unit; IQR: interquartile range [first and third quartile]; P_a_CO_2_: partial pressure arterial carbon dioxide; P_a_O_2_: partial pressure arterial oxygen; PEEP: positive end-expiratory pressure; PIP: peak inspiratory pressure; SD: standard deviation; SOFA: sequential organ failure assessment.*

**Table S3.** Adjunctive measures performed during ICU stay in patients with treatment limitations at ICU discharge stratified by vital status at hospital discharge.

| **Parameter** | **Alive**  **N=118** | **Dead**  **N=77** | **Total**  **N=195** | *p-value* |
| --- | --- | --- | --- | --- |
| Neuromuscular blockade, n (%) | 11 (9.32) | 1 (1.30) | 12 (6.15) | ***0.0299*** |
| Recruitment maneuvers, n (%) | 13 (11.02) | 6 (7.79) | 19 (9.74) | *0.4579* |
| Prone positioning, n (%) | 1 (0.85) | 1 (1.30) | 2 (1.03) | *1.0000* |
| ECMO, n (%) | 0 (0.00) | 0.(0.00) | 0 (0.00) | *-* |
| Inhaled vasodilators, n (%) | 8 (6.78) | 1 (1.30) | 9 (4.62) | *0.0907* |
| HFOV, n (%) | 2 (1.69) | 1 (1.30) | 3 (1.54) | *1.0000* |
| None of above adjunctive measures, n (%) | 91 (77.12) | 68 (88.31) | 159 (81.54) | ***0.0489*** |

*Abbreviations: ECMO: extra corporeal membrane oxygenation; HFOV: high frequency oscillatory ventilation.*

**Table S4.** Ventilator setting in patients with treatment limitations at ICU discharge who received invasive MV for at least 2 days (from AHRF onset) stratified by vital status at hospital discharge.

| **Parameter** | **Alive**  **N=118** | **Dead**  **N=77** | **Total**  **N=195** | *p-value* |
| --- | --- | --- | --- | --- |
| Patients on IMV at 1^st^ and 2^nd^ day, n (%) | **64 (54.24)** | **50 (64.94)** | **114 (58.46)** | *0.1384* |
| Last day on IMV (*with collected data*), median [IQR] | 7.0 [3.0-14.0] | 5.0 [3.0-7.0] | 7.0 [3.0-10.0] | ***0.0257*** |
| Non-Invasive Mechanical Ventilation after IMV, n (%) | 7 (10.94) | 5 (10.00) | 12 (10.53) | *0.8714* |
| **Ventilator setting at 1^st^ day of IMV** |  |  |  |  |
| Controlled ventilation, n (%) | 32 (50.79) | 38 (76.00) | 70 (61.95) | ***0.0061*** |
| FiO_2_, median [IQR] | 0.50 [0.40-0.70] | 0.50 [0.40-0.70] | 0.50 [0.40-0.70] | *0.3280* |
| Set respiratory rate (breaths/min), mean ± SD | 17.54 ± 4.70 | 16.24 ± 4.90 | 16.93 ± 4.82 | *0.1699* |
| Total respiratory rate (breaths/min), mean ± SD | 20.13 ± 4.99 | 19.02 ± 6.67 | 19.64 ± 5.79 | ***0.0335*** |
| Tidal volume (ml/kg IBW), mean ± SD | 7.87 ± 2.00 | 7.97 ± 1.89 | 7.91 ± 1.94 | *0.6997* |
| High tidal volume (>8 ml/kg IBW), n (%) | 19 (32.20) | 22 (44.90) | 41 (37.96) | *0.1759* |
| Dynamic compliance (ml/cmH_2_O), mean ± SD | 29.60 ± 15.09 | 29.64 ± 16.84 | 29.62 ± 15.82 | *0.8813* |
| PEEP (cmH_2_O), mean ± SD | 7.73 ± 2.60 | 7.02 ± 2.41 | 7.42 ± 2.53 | *0.0988* |
| PIP (cmH_2_O), mean ± SD | 26.03 ± 7.56 | 26.22 ± 8.54 | 26.12 ± 7.98 | *0.7338* |
| Plateau pressure measured, n (%) | 14 (21.88) | 13 (26.00) | 27 (23.68) | *0.6072* |
| Plateau pressure (cmH_2_O), mean ± SD | 21.57 ± 3.99 | 20.77 ± 3.96 | 21.19 ± 3.92 | *0.6052* |
| Driving pressure (cmH_2_O), mean ± SD | 14.29 ± 3.99 | 14.08 ± 4.65 | 14.19 ± 4.23 | *0.9011* |
| Minute ventilation (l/min), mean ± SD | 9.22 ± 3.07 | 8.74 ± 2.84 | 9.01 ± 2.97 | *0.2728* |
| Standardized minute ventilation (l/min), mean ± SD | 10.56 ± 4.68 | 9.89 ± 4.53 | 10.26 ± 4.61 | *0.2226* |
| **Ventilator setting at last available day in IMV in ICU** |  |  |  |  |
| Controlled ventilation, n (%) | 7 (11.11) | 15 (30.61) | 22 (19.64) | ***0.0100*** |
| FiO_2_ |  |  |  |  |
| Median [IQR] | 0.40 [0.30-0.40] | 0.35 [0.30-0.40] | 0.36 [0.30-0.40] | *0.9728* |
| Available data, n (%) | 59 (92.19) | 50 (100.00) | 109 (95.61) | *0.0664* |
| Set respiratory rate (breaths/min) |  |  |  |  |
| Mean ± SD | 16.64 ± 8.50 | 14.88 ± 6.39 | 15.93 ± 7.72 | *0.3903* |
| Available data, n (%) | 36 (56.25) | 24 (48.00) | 60 (52.63) | *0.3814* |
| Total respiratory rate (breaths/min) |  |  |  |  |
| Mean ± SD | 21.89 ± 5.85 | 19.46 ± 5.54 | 20.82 ± 5.82 | *0.0571* |
| Available data, n (%) | 61 (95.31) | 48 (96.00) | 109 (95.61) | *1.0000* |
| Tidal volume (ml/kg IBW) |  |  |  |  |
| Mean ± SD | 7.77 ± 2.01 | 7.88 ± 2.38 | 7.82 ± 2.19 | *0.8695* |
| High tidal volume (>8 ml/kg IBW), n (%) | 24 (44.44) | 19 (39.58) | 43 (42.16) | *0.6197* |
| Available data, n (%) | 54 (84.38) | 48 (96.00) | 102 (89.47) | ***0.0448*** |
| Dynamic compliance (ml/cmH_2_O) |  |  |  |  |
| Mean ± SD | 44.29 ± 42.67 | 45.76 ± 31.53 | 44.95 ± 37.89 | *0.2971* |
| Available data, n (%) | 55 (85.94) | 45 (90.00) | 100 (87.72) | *0.5120* |
| PEEP (cmH_2_O) |  |  |  |  |
| Mean ± SD | 6.22 ± 2.04 | 5.98 ± 2.07 | 6.11 ± 2.05 | *0.6268* |
| Available data, n (%) | 60 (93.75) | 50 (100.00) | 110 (96.49) | *0.1297* |
| PIP (cmH_2_O) |  |  |  |  |
| Mean ± SD | 20.71 ± 8.30 | 19.38 ± 6.47 | 20.13 ± 7.55 | *0.3226* |
| Available data, n (%) | 58 (90.63) | 45 (90.00) | 103 (90.35) | *1.0000* |
| Plateau pressure (cmH_2_O) |  |  |  |  |
| Mean ± SD | 18.67 ± 2.31 | 20.00 ± 4.75 | 19.64 ± 4.15 | *0.6599* |
| Available data, n (%) | 3 (4.69) | 8 (16.00) | 11 (9.65) | *0.0566* |
| Driving pressure (cmH_2_O) |  |  |  |  |
| Mean ± SD | 12.67 ± 2.08 | 14.25 ± 2.05 | 13.82 ± 2.09 | *0.2854* |
| Available data, n (%) | 3 (4.69) | 8 (16.00) | 11 (9.65) | *0.0566* |
| Minute ventilation (l/min) |  |  |  |  |
| Mean ± SD | 9.79 ± 2.95 | 8.86 ± 3.21 | 9.37 ± 3.09 | *0.1214* |
| Available data, n (%) | 59 (92.19) | 48 (96.00) | 107 (93.86) | *0.4641* |
| Standardized minute ventilation (l/min) |  |  |  |  |
| Mean ± SD | 10.53 ± 3.65 | 8.58 ± 3.01 | 9.66 ± 3.50 | ***0.0251*** |
| Available data, n (%) | 52 (81.25) | 42 (84.00) | 94 (82.46) | *0.7017* |

*Abbreviations: FiO_2_: fraction of inspired oxygen; IBW: ideal body weight; IMV: invasive mechanical ventilation; IQR: interquartile range [first and third quartile]; PEEP: positive end-expiratory pressure; PIP: peak inspiratory pressure; SD: standard deviation.*

**Table S5.** Factors associated to hospital mortality in patients with treatment limitations at ICU discharge.

|  | **OR (95% CI)** | **p-value** |
| --- | --- | --- |
| **Multivariable logistic regression model 1 (n=116 on 195)** |  |  |
| PaO_2_ / FiO_2_ (10 mmHg) | 0.952 (0.914 - 0.991) | 0.0166 |
| Clinical recognition of ARDS during ICU stay (ref. No) | 0.350 (0.146 - 0.839) | 0.0185 |
| **Multivariable logistic regression model on patients on IMV for at least 2 days (from AHRF onset) (n=109 on 114)** |  |  |
| Total respiratory rate (breath / min) | 0.916 (0.851 - 0.986) | 0.0203 |
| Adjunctive measures during ICU stay (ref. No) | 0.328 (0.125 - 0.860) | 0.0235 |

*Abbreviations: CI: confidence interval; FiO_2_: fraction of inspired oxygen; ICU: intensive care unit; OR: odds ratio; P_a_O_2_: partial pressure arterial oxygen.*

*Note 1: model 1 was identified by stepwise approach using as possible predictors: baseline patients’ characteristics, geo-economic area of enrollment, parameters of illness of severity at last available day in ICU, use of adjunctive measures during ICU stay and ICU characteristics.*

*Note 2: model 2 was identified by stepwise approach using as possible predictors: baseline patients’ characteristics, geo-economic area of enrollment, parameters of illness of severity and ventilator setting at last available day in ICU, use of adjunctive measures during ICU stay and ICU characteristics.*
